# Loss of Pex1 in Inner Ear Hair Cells Contributes to Cochlear Synaptopathy and Hearing Loss

**DOI:** 10.3390/cells11243982

**Published:** 2022-12-09

**Authors:** Stephanie A. Mauriac, Thibault Peineau, Aamir Zuberi, Cathleen Lutz, Gwénaëlle S. G. Géléoc

**Affiliations:** 1 Department of Otolaryngology, Boston Children’s Hospital, Boston, MA 02115, USA; 2 Kirby Neurobiology Center, Harvard Medical School, Boston, MA 02115, USA; 3 Rare Disease Translational Center, The Jackson Laboratory, Bar Harbor, ME 04609, USA; 4 Technology Evaluation and Development Research Laboratory, The Jackson Laboratory, Bar Harbor, ME 04609, USA

**Keywords:** Pex1, hearing loss, peroxisome, synapse, hair cells, PBD-ZSD

## Abstract

Peroxisome Biogenesis Disorders (PBD) and Zellweger syndrome spectrum disorders (ZSD) are rare genetic multisystem disorders that include hearing impairment and are associated with defects in peroxisome assembly, function, or both. Mutations in 13 peroxin (*PEX*) genes have been found to cause PBD-ZSD with ~70% of patients harboring mutations in *PEX1*. Limited research has focused on the impact of peroxisomal disorders on auditory function. As sensory hair cells are particularly vulnerable to metabolic changes, we hypothesize that mutations in *PEX1* lead to oxidative stress affecting hair cells of the inner ear, subsequently resulting in hair cell degeneration and hearing loss. Global deletion of the *Pex1* gene is neonatal lethal in mice, impairing any postnatal studies. To overcome this limitation, we created conditional knockout mice (cKO) using *Gfi1^Cre^* or *VGlut3^Cre^* expressing mice crossed to floxed *Pex1* mice to allow for selective deletion of *Pex1* in the hair cells of the inner ear. We find that *Pex1* excision in inner hair cells (IHCs) leads to progressive hearing loss associated with significant decrease in auditory brainstem responses (ABR), specifically ABR wave I amplitude, indicative of synaptic defects. Analysis of IHC synapses in cKO mice reveals a decrease in ribbon synapse volume and functional alterations in exocytosis. Concomitantly, we observe a decrease in peroxisomal number, indicative of oxidative stress imbalance. Taken together, these results suggest a critical function of *Pex1* in development and maturation of IHC-spiral ganglion synapses and auditory function.

## 1. Introduction

Peroxisomal Biogenesis and Zellweger Spectrum Disorders (PBD-ZSD) are a group of rare autosomal recessive disorders caused by mutations in *PEX *genes, characterized by defective peroxisome assembly and function. Patients with PBD-ZSD display physiological, developmental, and neurological complications that include visual and sensorineural hearing loss (SNHL) [1,2,3]. Based on genotype–phenotype correlations, PBD-ZSD are classified as severe, intermediate, or mild within the PBD-ZSD spectrum [4]. Severe forms of the disease are associated with a complete loss of peroxisomal function. Infants with severe forms of PBD-ZSD display severe impairments at birth and are associated with a short life expectancy, typically under a year. Milder forms of the disease comprise missense mutations, are typically associated with milder slowly progressing phenotypes and lead to variable life expectancy up to adulthood [5]. While drugs have been developed that can partially alleviate the symptoms of the disease, there is currently no cure for PBD-ZSD.

In mammalian cells, peroxisomes play a key role in anabolic (biosynthetic) and catabolic (degradative) pathways [6]. These organelles are indispensable for lipid metabolism (i.e., biosynthesis of etherphospholipids, fatty acid alpha/beta-oxidation, bile acid and docosahexaenoic acid, DHA) but also may serve as protective organelles by playing a role in detoxification of reactive oxygen species (ROS) [7].

The biogenesis of peroxisomes depends on different peroxins, or PEX proteins, which play a role in the targeting of the peroxisomal membrane proteins (PMP) to the peroxisome membrane, import of peroxisomal matrix proteins and peroxisomal proliferation which occurs in response to external cues [8]. Mutations in thirteen *PEX* genes have been found to cause PBD-ZSD [4,9,10,11]. Mutations in the *PEX1* gene, encoding for the peroxisomal biogenesis factor 1, are the most common, found in nearly 70 percent of affected individuals [1,12,13]. The most common *PEX1* mutation is *hPEX1.G843D*.

PBD-ZSD patients suffer from moderately severe to profound hearing loss [3]. Patients with the common *G843D *mutation in one of the *PEX1* alleles along with a null allele (*G843D*/null) suffer from severe hearing loss with thresholds ranging 70 to 100 decibels (dB) sound-pressure level (SPL). Patients who possess homozygous mutations (*G843D/G843D*) suffer from moderate to profound hearing loss with threshold ranging 50 to 100 dB SPL [3].

SNHL most commonly arises from damage or loss of sensory hair cells, hair cell-neuronal synapse or degeneration of neurons. Sensory hair cells and spiral ganglion neurons are particularly vulnerable to various stresses including oxidative stress. Even though a recent study demonstrated a non-canonical function of peroxisomes that leads to a better resistance of some cells to oxidative stress [14], we hypothesized that *PEX1* mutations alter sensory hair cell stability and survival leading to progressive SNHL in patients partly due to the increase of oxidative stress, the severe decrease of plasmalogen synthesis [15] and defect in the import of antioxidant enzymes.

To assess how *PEX1* mutations affect hair cells (HCs) we took advantage of different conditional knockout mouse models and assessed physiology and morphology of the auditory organ. Our work demonstrates that IHCs are vulnerable to the loss of Pex1 protein which leads to alteration of synapses and progressive hearing loss.

## 2. Materials and Methods

### 2.1. Animals

All animal experiments were performed in accordance with the NIH guidelines and were approved by the Institutional Animal Care and Use Committee (protocols #20-02-4149R and #00001240) at Boston Children’s Hospital.

**Figure 1:**
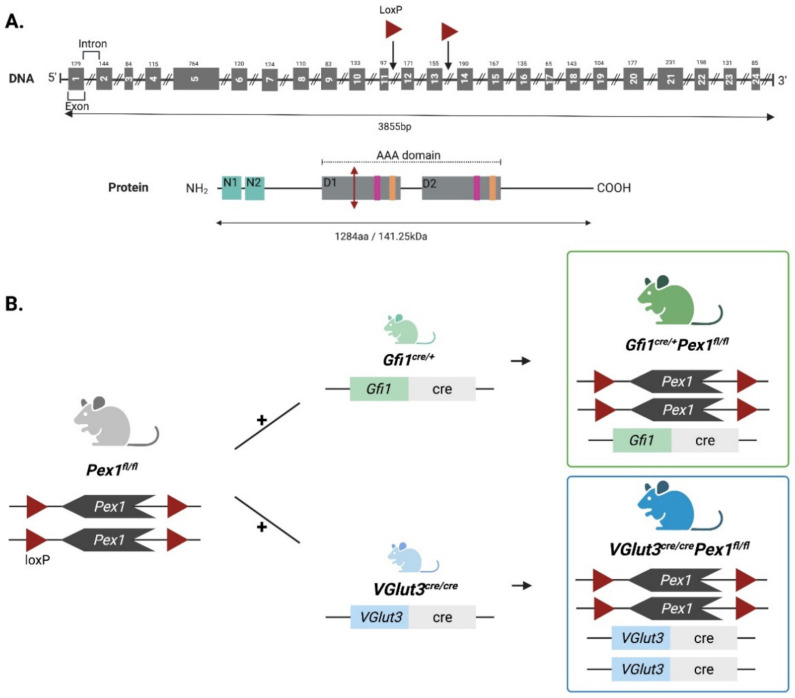
Generation of new organ specific *Pex1* knockout mice. (**A**) Schematic of *Pex1* gene and Pex1 protein. LoxP sites (red triangle) were inserted inside the introns 11 and 13 of the *Pex1* gene. Excision of exons 12 and 13, upon *Cre* recombination, leads to the production of a truncated protein (red vertical arrow). The Pex1 protein is composed of two N-terminal domains (N1 and N2) and two functional AAA domains (D1 and D2). The conserved Walker motifs domains into AAA domains are indicated (Walker A motifs: magenta and Walker B motifs: orange); (**B**) Breeding scheme for generating *Gfi1^cre/+^Pex1^fl/fl^* mice line (*Cre* expressed in inner and outer hair cells around E15.5) and *VGlut3^cre/cre^Pex1^fl/fl^* mouse line (*Cre* expressed in inner hair cells around birth). *Pex1^fl/fl^* mice were used as a control. Created with BioRender.com (Accessed date: 6 December 2022).

Male and female *Pex1^fl/fl^* were obtained as a private strain from The Jackson Laboratory (JR 32722). These mice were generated from the *Pex1-G844D *mice (*B6.Cg-Pex1^tm1.1Sjms^/Mmjax; RRID:MMRRC_037405-JAXInfo*) which include loxP sites flanking exons 12 and 13 (Figure 1) and was edited to correct for the *G844D* mutation and bring it back to the wild type sequence. The *Gfi1^Cre^* knock-in mice were generated by Dr. Lin Gan at the University of Rochester [16] and were provided for this study by Dr. Jian Zuo at St Jude Children’s Research Hospital. These mice can only be bred with one copy of the *Cre* driver. *Slc17a8-ires-Cre* (referred to as *VGlut3^Cre^*) knock-in mice were obtained at Bradford Lowell/BIDMC [17]. Mice were maintained in a C57BL/6J background in our facility at Boston Children’s Hospital. Mice of both sexes were used in similar numbers. In this case, the *Cre *insertion does not affect endogenous expression of *VGlut3* and the mice can be bred to have two copies of the *Cre* allele. Breeding was carried out to obtain *Cre* expression under homozygous *Pex1* floxed alleles with one copy of the *Cre* allele (*Gfi1^Cre/+^*) for *Gfi1^Cre^* expressing mice, and two copies (*VGlut3^Cre/Cre^*) for *VGlut3^Cre^* expressing mice. Tissue collection was performed immediately post-mortem.

### 2.2. Genotyping

Genotyping was performed by Polymerase Chain Reaction (PCR) using GoTaq^®^ Master Mix (Promega #M7122) using primer sets included in Table 1. For the *Pex1^fl/fl^* genotyping, we followed the protocol designed for the *B6.Cg-Pex1^tm1.1Sjms^/Mmja *mice by The Jackson Laboratory. For the *Gfi1^Cre^* genotyping, the PCR reaction was run in a thermocycler programmed for 94 °C for 3 min, [94 °C for 30 s, 61 °C for 1 min, 72 °C for 45 s] × 30 cycles, 72 °C for 5 min, and then maintained at 4 °C until further analysis. For the *VGlut3^Cre^* genotyping, the PCR reaction was run in a thermocycler programmed for 94 °C for 5 min [94 °C for 1 min, 55 °C for 30 s, 72 °C for 1 min] × 30 cycles, 72 °C for 5 min, and then maintained at 4 °C until further analysis. A 1% agarose gel was used to analyze PCR products on iBright CL1500 imaging system.

### 2.3. Electrophysiology

ABRs (auditory brainstem recordings) and DPOAEs (distortion-product otoacoustic emissions) recordings were performed as previously described [18] from 5.6 to 32.0 kHz at 1 and 4 months on control *Pex1^fl/fl^*, as well as *Gfi1^Cre/+^Pex1^fl/fl^*; and *VGlut3^Cre/Cre^Pex1^fl/fl^* mice. ABR wave I amplitude was determined from positive peak 1 (P1) to negative peak 1 (N1) and analyzed from 5.6 to 32.0 kHz at 1 and 4 months in control *Pex1^fl/fl^*, as well as *Gfi1^Cre/+^Pex1^fl/fl^*; and *VGlut3^Cre/Cre^Pex1^fl/fl^* mice. ABR wave IV amplitude was determined from positive peak 4 (P4) to negative peak 4 (N4) and analyzed from 5.6 to 32.0 kHz at 1 and 4 months in control *Pex1^fl/fl^*, and *VGlut3^Cre/cre^Pex1^fl/fl^* mice. ABR wave I and IV latency reflect the distance from the origin to positive peak 1 (P1) or positive peak 4 (P4), respectively.

Whole cell electrophysiological recordings were performed on IHCs from acutely dissected apical cochlear explants from post natal (P)18-25 mice as described previously [19]. In brief, temporal bones were extracted from mice euthanized with CO_2_. Explants were placed in cold (4 °C) extracellular perilymph-like solution containing: NaCl, 135 mM; KCl, 5.8 mM; CaCl_2,_ 1.3 mM; MgCl_2,_ 0.9 mM; NaH_2_PO_4,_ 0.7 mM; Glucose, 5.6 mM; Na pyruvate, 2 mM; HEPES, 10 mM; pH 7.4 (adjusted with 5N NaOH); 305 mOsm (adjusted with sodium chloride). The mid-apical turn (20–40% of the apex), an area coding for frequencies ranging from 8 to 16 kHz [20], was dissected out from the explant [19,21,22]. The explants were placed under a pin in the recording chamber after which the tectorial membrane was removed. The preparation was mounted on a Zeiss Axioskop 2FS upright microscope (Zeiss, Oberkochen, Germany) and viewed with a Zeiss Achroplan 63X immersion lens (Zeiss, Oberkochen, Germany). Extracellular recording solutions were complemented with Apamin, 0.25 μM (Tocris Bioscience; ref #1652) and XE-991, 1 μM (Sigma-Aldrich; ref #X2254) to block SK channels and KCNQ4 channels, respectively. All experiments were performed at room temperature (18–22 °C) not exceeding 2 h after the dissection. Potassium (K^+^), calcium (Ca^2+^) currents and membrane capacitance were recorded using an EPC10 amplifier controlled by Patchmaster pulse software (HEKA Elektronik, Germany). Patch pipettes were pulled with a laser micropipette puller (P-2000, Sutter Instrument, Novato, CA, USA) and fire-polished with a microforge (MF-2000, World Precision Instruments, Sarasota, FL, USA) to obtain a resistance ranging 2 to 3 MΩ. Recording pipettes were filled with a KCl-based intracellular solution containing: KCl, 158 mM; MgCl_2_, 2 mM; EGTA, 1.1 mM; HEPES, 5 mM; and KOH, 3.05 mM; pH 7.2 (adjusted with 1 M KOH); 295 mOsm (adjusted with sucrose) for K^+^ currents recordings and with a cesium-based intracellular solution containing: CsCl, 145 mM; MgCl2, 1 mM; HEPES, 5 mM; EGTA, 1 mM; TEA, 20 mM; ATP, 2 mM; GTP 0.3 mM; pH 7.2 (adjusted with 1 M CsOH); 300 mOsm (adjusted with sucrose) for Ca^2+^ currents recordings.

Measurements of the resting membrane capacitance (cell size) of IHCs were obtained in whole-cell voltage-clamp configuration at −70 mV and after 2 min equilibrium of the internal patch-pipette recording solution with the IHC cytosol as described previously [19]. K^+^ recordings were obtained in whole-cell voltage-clamp configuration with 20 ms and 500 ms depolarizing steps from −90 mV to +70 mV with a 10 mV increment. Membrane potentials were corrected offline for the residual series resistance Rs as described previously [23]. Ca^2+^ IV ramp curves were elicited in whole-cell voltage-clamp configuration with a depolarizing step from −80 mV to +10 mV increasing 1 mV every millisecond during 90 ms. Kinetics of the fast exocytosis of the readily releasable pool (RRP) of vesicles were evoked by depolarizing voltage-steps from −80 mV to −10 mV (corresponding to the maximum inward Ca^2+^ current) with an increasing duration from 5 ms to 80 ms. Simple sustained exocytosis was obtained by 100 ms depolarizing voltage-steps from −80 mV to −10 mV.

### 2.4. Western Blots

At 8 weeks of age, liver tissue from control *Pex1^fl/fl^*, *Gfi1^Cre/+^Pex1^fl/fl^*, and *VGlut3^Cre/Cre^Pex1^fl/fl^* mice were rapidly harvested and snapped frozen into liquid nitrogen. Samples were lysed into RIPA lysis and extraction buffer (Thermofisher scientific, ref #89901) supplemented with protease inhibitor (Millipore Sigma, Sigmafast protease inhibitor, ref #S8830). After manual homogenization, samples were incubated for 3 h at 4 °C on a rocker. Supernatant was extracted after a 14,000 rpm centrifugation for 10 min at 4 °C. Protein concentration was measured with a BCA protein assay kit (Thermofisher scientific, ref #23227) on a nanodrop 2000 spectrophotometer. After resuspension in 4X SDS sample buffer (Biorad, ref #1610747), each sample was diluted to 20 µg/µL. Samples were denatured at 95 °C for 5 min. Lysates were subjected to SDS–PAGE on 4–15% SDS–polyacrylamide gel electrophoresis (Biorad, ref #456808) and transferred to hydrophobic polyvinylidene difluoride transfer membrane (Millipore Sigma, ref #IPSN07852). Antibodies were used to visualize Pex1 protein (Pex1 rabbit polyclonal antibody, 1:100, Proteintech, ref #13669), peroxisomal membrane proteins (PMP70 rabbit polyclonal antibody, 1:100, Abcam, ref #ab3421), and β-Actin HRP (1:2000, Santa Cruz, ref #SC47778-HRP) which was used to normalize protein levels for each set. Horseradish peroxidase (HRP)—conjugated anti-rabbit immunoglobulin G (1:5000; Millipore Sigma, ref #NA934) was used as a secondary antibody. For chemiluminescence reaction Amersham ECL Prime Western Blotting Detection Reagent (cytiva, ref #RPN2232) was used for visualization on a densitometric (iBright CL1500 imaging system). Each band was quantified using the ‘Analyze > Gel’ function on Fiji software. Each band was normalized to β-Actin level. Each experiment was carried out at least three times.

### 2.5. Immunostaining and Synapse Analysis

Immunostaining and synapse analysis were performed as described previously [24]. In brief, cochlea of 4 months control *Pex1^fl/fl^*, *Gfi1^Cre/+^Pex1^fl/fl^*; and *VGlut3^Cre/Cre^Pex1^fl/fl^* mice were fixed with 4% PFA for 1 h at room temperature, decalcified, and dissected for whole-mount processing. Tissues were permeabilized and blocked with normal horse serum (NHS) (5%, Jackson ImmunoResearch, ref #008-000-121) and triton (0.3%, Sigma Aldrich, ref #X100-5ML) for 1 h at room temperature. Then, cochleas were immuno-stained with anti-CtBP2 (pre-synaptic marker, IgG1, 1:500, BD Biosciences, ref #BDB612044), GluR2 (Post-synaptic, IgG2a 1:500 Millipore Sigma, ref #MABN1189), Myosin7a (HC marker, 1:500, Proteus Biosciences, ref #25-6790) overnight at 37 °C. After PBS wash, cochleas were incubated at 37 °C for 3 h with fluorophore-conjugated donkey anti-rabbit secondary antibody 647 (1:500, Alexa Fluor Thermofisher, ref #A31573), fluorophore-conjugated goat anti-mouse IgG2a 488 secondary antibody (1:500, Alexa Fluor Thermofisher, ref #A21131), fluorophore-conjugated goat anti-mouse IgG1 546 secondary antibody (1:500, Alexa Fluor Thermofisher, ref #A21123), and Phalloidin 405 (1:500, Thermofisher, ref #A30104). After PBS wash, cochleas were mounted with Vectashield antifade mounting medium containing DAPI (Vector laboratories, ref #H-1200-10). Images were acquired using the Zeiss LSM800 confocal microscope. Z-stacks were obtained from 5.6 to 32.0 kHz regions with 0.3 μm step. Frequency maps were designed by measuring the apex-to-base length by using the ‘measure line’ plugin on Fiji software. The number and the volume of ribbon synapses per IHC were done using the function ‘spots’ and ‘volumes’ on Imaris Cell imaging software (Oxford Instruments).

### 2.6. Data Analysis and Statistics

ABRs and ribbon synapses data were analyzed with Prism—GraphPad version 9 software or OriginPro 2022b software (OriginLab, Northampton, MA, USA). For Western blot analysis, results were analyzed with Prism—GraphPad version 9 software. For K^+^ and Ca^2+^ currents recordings, capacitance measurement in IHCs, and exocytosis analysis results were analyzed with OriginPro 2022b software (OriginLab, Northampton, MA, USA). Statistical analyses with two data sets were made by two-tailed unpaired *t*-tests or for comparisons of more than two data sets, one-way ANOVA or two-way ANOVA followed by a Tukey mean comparison test. All results are expressed as mean ± S.E.M.

### 2.7. Resources

All resources used for this project are summarized in Table 2.

## 3. Results

### 3.1. Generation and Characterization of Two New Organ-Specific Pex1 Knockout Mouse Lines

We generated two novel mouse models with conditional deletion of *Pex1 *in the inner ear to determine how deletion of Pex1 proteins affects the auditory organ and specifically sensory hair cells (HCs). For this purpose, we took advantage of *Pex1* floxed (*Pex1^fl/fl^*) mouse line developed at The Jackson Laboratory that possess the wild type *Pex1* allele with LoxP sites localized in introns 11 and 13 (Figure 1A). The expression of the *Cre*-recombinase leads to the excision of the exons 12 and 13. *Pex1^fl/fl^* mice were crossed to HC-specific *Cre* expressing mice to conditionally delete *Pex1* in HCs of the inner ear. We used two *Cre*-driver strains, *Gfi1^Cre^* and *VGlut3^Cre^* mice. *Gfi1^Cre^* mouse line was used to excise *Pex1* gene in all HCs at early stages (~embryonic stage (E) 15.5), while *VGlut3^Cre^* mouse line was used to excise *Pex1* gene specifically in IHCs around birth (Figure 1B). *Pex1^fl/fl^* mice were crossed to *Gfi1^Cre^* mice to produce *Gfi1^Cre/+^Pex1^fl/+^* mice. These mice were further crossed to obtain *Gfi1^Cre/+^Pex1^fl/fl^* mice. In parallel, *Pex1^fl/fl^* mice were crossed to *VGlut3^Cre^* mice to obtain *VGlut3^Cre/+^Pex1^fl/fl^* mice (with one copy of the *Cre*-expressing allele) and *VGlut3^Cre/Cre^Pex1^fl/fl^* mice (with two copies of the *Cre*-expressing allele). The homozygous *VGlut3^Cre^* mice were generated to increase the efficiency of the recombination. Contrary to homozygous *Gfi1^Cre^* mice, *VGlut3^Cre^* mice can be bred to possess two copies of the *Cre* allele which does not disrupt *VGlut3* expression [17]. All genotypes were analyzed but only results for *Pex1^fl/fl^*, *Gfi1^Cre/+^Pex1^fl/fl^, and VGlut3^Cre/Cre^Pex1^fl/fl^* are presented in this report. *Cre* recombination in *Gfi1^Cre/+^Pex1^fl/fl^* and *VGlut3^Cre/Cre^Pex1^fl/fl^* mice was validated by RT-qPCR (data not shown) and Western blot in liver samples, which also express *Gfi1* and *VGlut3* (Figure S1A–D; Table S16). No obvious changes in gross tissue morphology were detected in the inner ear of the conditional mouse models, which displayed normal hair cell organization, stereocilia bundle shape and length as observed on whole mount cochleas from *Pex1^fl/fl^, Gfi1^Cre/+^Pex1^fl/fl^* and *VGlut3^Cre/Cre^Pex1^fl/fl^* mouse lines (data not shown). Breeding of the lines took three generations to obtain the experimental *Cre* expressing mice in presence of floxed allele and absence of wild type *Pex1 *allele. This model provides a unique opportunity to analyze *Pex1* gene function in the inner ear.

### 3.2. Gfi1^Cre/+^Pex1^fl/fl^ Mice Show Normal Hearing but Reduced Wave I Amplitude

**Figure 2:**
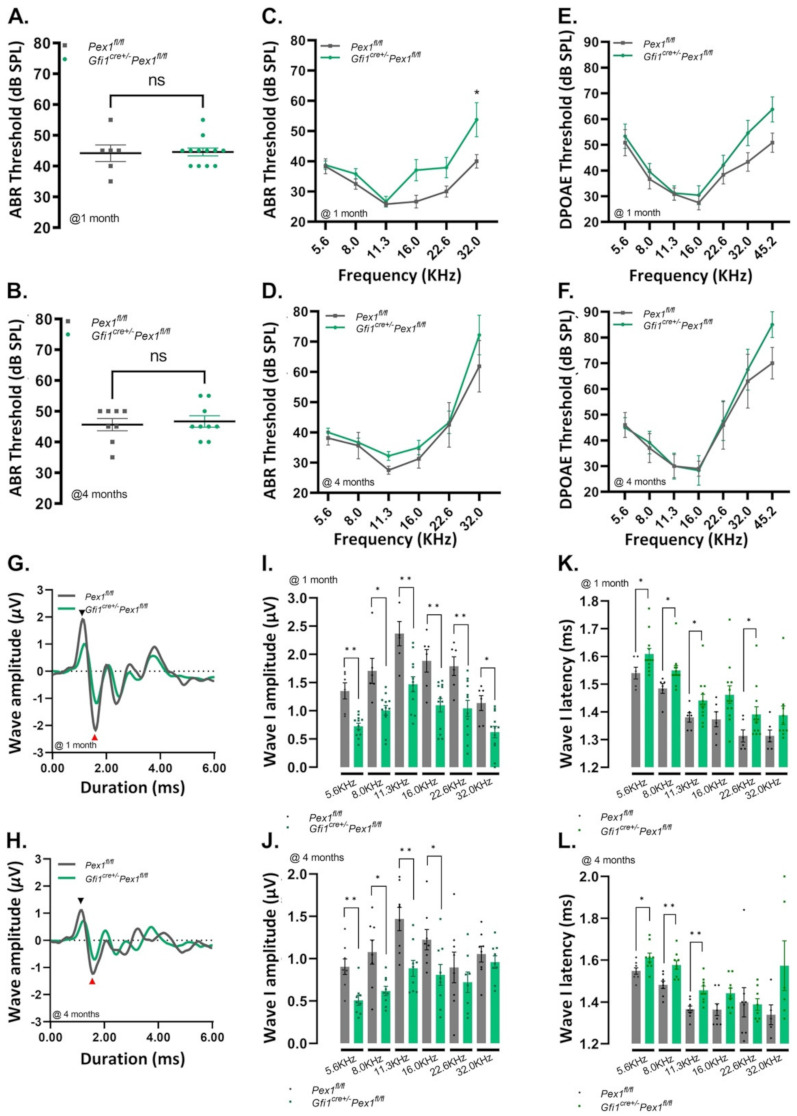
Decrease of the wave I ABR amplitude is observed in *Gfi1^cre/+^Pex1^fl/fl^* mice. (**A**,**B**) Click ABR recordings on *Pex1^fl/fl^* (grey) and *Gfi1^cre/+^Pex1^fl/fl^* (green) mice at 1 month (Table S1; mean ± S.E.M. ns p = 0.9334, Mann–Whitney test) (**A**) and 4 months (Table S1; mean ± S.E.M. ns p = 0.9638, Mann–Whitney test) (**B**); (**C**,**D**) Pure-tone ABR recordings as a function of frequencies (kHz) on *Pex1^fl/fl^* (grey) and *Gfi1^cre/+^Pex1^fl/fl^* (green) mice at 1 month (Table S1; mean ± S.E.M. 5.6 kHz: ns *p* > 0.999, 8.0 kHz: ns *p* > 0.999, 11.3 kHz: ns *p* > 0.999, 16.0 kHz: ns *p* = 0.213, 22.6 kHz: ns *p* = 0.631, 32.0 kHz: * *p* = 0.033—2way ANOVA Bonferroni’s multi-comparison test) (**C**) and 4 months (Table S1; mean ± S.E.M. 5.6kHz: ns *p* > 0.999, 8.0 kHz: ns *p* > 0.999, 11.3 kHz: ns *p* > 0.999, 16.0 kHz: ns *p* > 0.999, 22.6 kHz: ns *p* > 0.999, 32.0 kHz: ns P = 0.551—2way ANOVA Bonferroni’s multi-comparison test) (**D**); (**E**,**F**) Distortion-product otoacoustic emissions (DPOAEs) recordings as a function of frequencies (kHz) on *Pex1^fl/fl^* (grey) and *Gfi1^cre/+^Pex1^fl/fl^* (green) mice at 1 month (Table S2; mean ± S.E.M. 5.6 kHz: ns *p* > 0.999, 8.0 kHz: ns *p* > 0.999, 11.3 kHz: ns *p* > 0.999, 16.0 kHz: ns *p* > 0.999, 22.6 kHz: ns *p* > 0.999, 32.0 kHz: ns *p* = 0.557, 45.2 kHz: ns *p* = 0.312—2way ANOVA Bonferroni’s multi-comparison test) (**E**) and 4 months (Table S2; mean ± S.E.M. 5.6kHz: ns *p* > 0.999, 8.0 kHz: ns *p* > 0.999, 11.3 kHz: ns p > 0.999, 16.0 kHz: ns *p* > 0.999, 22.6 kHz: ns *p* > 0.999, 32.0 kHz: ns *p* > 0.999, 45.2 kHz: ns *p* = 0.300—2way ANOVA Bonferroni’s multi-comparison test) (**F**); (**G**,**H**) Average of individual click ABR wave traces recorded on *Pex1^fl/fl^* (grey) and *Gfi1^cre/+^Pex1^fl/fl^* (green) mice at 1 month (**G**) and 4 months (**H**). Black arrowhead indicates the positive peak of wave I (P1) and the red arrowhead indicates the negative peak of the wave I (N1); (**I**,**J**) Average of highest intensity tested (90, 80, 70 dB SPL) of wave I amplitude (N1-P1) on pure tones recording from 5.6 to 32.0 kHz at 1 month (Table S3; mean ± S.E.M. 5.6 kHz: ** *p* = 0.0054, 8.0 kHz: * *p* = 0.0235, 11.3 kHz: ** *p* = 0.0064, 16.0 kHz: ** *p* = 0.0084, 22.6 kHz: ** *p* = 0.0049, 32.0 kHz: * *p* = 0.0109—unpaired *t*-test with Welch’s correction); (**I**) and 4 months (Table S3; mean ± S.E.M. 5.6 kHz: ** *p* = 0.0034, 8.0 kHz: * *p* = 0.0144, 11.3 kHz: ** *p* = 0.0042, 16.0 kHz: * *p* = 0.0293, 22.6 kHz: ns *p* = 0.4393, 32.0 kHz: ns *p* = 0.4436—unpaired *t*-test with Welch’s correction) (**J**) on *Pex1^fl/fl^* (grey) and *Gfi1^cre/+^Pex1^fl/fl^* (green) mice; (**K**,**L**) Measures of the wave I latency on pure tones recorded from 5.6 to 32.0 kHz at 1 month (Table S4; mean ± S.E.M. 5.6 kHz: * *p* = 0.0341, 8.0 kHz: * *p* = 0.0196, 11.3 kHz: * *p* = 0.0451, 16.0 kHz: ns *p* = 0.0502, 22.6 kHz: * *p* = 0.0417, 32.0 kHz: ns *p* = 0.0807—unpaired *t*-test with Welch’s correction) (**K**) and 4 months (Table S4; mean ± S.E.M. 5.6 kHz: * *p* = 0.0261, 8.0 kHz: ** *p* = 0.0034, 11.3 kHz: ** *p* = 0.0024, 16.0 kHz: ns *p* = 0.0547, 22.6 kHz: ns *p* = 0.8970, 32.0 kHz: ns *p* = 0.1122—unpaired *t*-test with Welch’s correction) (**L**) on *Pex1^fl/fl^* (grey) and *Gfi1^cre/+^Pex1^fl/fl^* (green) mice. Averages the highest intensity tested (90, 80, 70dB SLP); *1 month: Pex1^fl/fl^* (*n* = 6), *Gfi1^cre/+^Pex1^fl/fl^* (*n* = 10–12), 4 months—*Pex1^fl/fl^* (*n* = 8), *Gfi1^cre/+^Pex1^fl/fl^* (*n* = 6–9).

To determine how alteration in *Pex1* expression affects HCs, we evaluated the auditory phenotype of the conditional *Pex1* knockout model. To that end, we first assessed click-evoked auditory brainstem responses (ABRs) at two time points, 1 month and 4 months. The threshold corresponds to the lowest intensity of sound leading to a detectable wave form. At 1 month of age, we found similar thresholds in both *Gfi1^Cre/+^Pex1^fl/fl^* and control *Pex1^fl/fl^* mice, with an average of 44.58 ± 1.30 dB SPL (*n* = 12) and 44.17 ± 2.71 dB SPL (*n* = 6), respectively (Table S1; Figure 2A).

Similar results were observed at 4 months of age in *Gfi1^Cre/+^Pex1^fl/fl^* and *Pex1^fl/fl^* mice (*Pex1^fl/fl^*: 45.63 ± 1.99 dB, n = 8; *Gfi1^Cre/+^Pex1^fl/fl^*: 46.67 ± 1.86 dB, *n* = 9; Table S1; Figure 2B). ABRs were also assessed in response to pure tones, at frequencies ranging from 5.6 to 32.0 kHz. At 1 month of age, we observed a mild threshold elevation in the high frequency range (from 16.0 to 32.0 kHz) compared to control (Table S1; Figure 2C). At 4 months of age, no significant threshold difference was observed between *Gfi1^Cre/+^Pex1^fl/fl^* and *Pex1^fl/fl^* mice (Table S1; Figure 2D). Outer hair cell (OHCs) function was assessed by recording distortion products otoacoustic emissions (DPOAEs) in the control and conditional KO mice. *Gfi1^Cre/+^Pex1^fl/fl^* mice had normal DPOAEs thresholds compared to *Pex1^fl/fl^* mice at 1 and 4 months of age, suggesting there was no alteration of OHC function in the conditional mice (Table S2; Figure 2E,F). While these data suggest that *Gfi1^Cre/+^Pex1^fl/fl^* mice have a normal auditory phenotype, analysis of the ABR waveforms revealed alterations of the response amplitude, affecting all peaks for the two time points tested (Figure 2G,H). Wave I amplitude reflects sound evoked activity of the afferent fibers innervating the IHCs of the cochlea. Since wave I amplitude is a good indicator of the activity of the distal cochlear nerve, we measured wave I amplitude and latency in response to pure tone stimuli, at 1 and 4 months. To that end, we averaged the values for the three highest intensities tested (70, 80 and 90 dB SPL). We find that wave I amplitude (i.e., N1-P1) is significantly reduced at 1 and 4 months of age (Table S3; Figure 2I,J, *p* values between * *p* = 0.0293 and ** *p* = 0.0034).

We also performed wave I response growth analysis. For this analysis, we defined wave I slope by generating a linear regression curve of the wave I amplitude as a function of sound intensity. The data demonstrate significant change in slope values associated with the decrease in wave I amplitude in most of the frequencies tested in *Gfi1^Cre/+^Pex1^fl/fl^* at 1 and 4 months (Figure S2A–L; Table S17; *p* value between * *p* = 0.0270 and *** *p* = 0.0005). In addition, wave I latency was slightly increased in *Gfi1^Cre/+^Pex1^fl/fl^* mice at 1 and 4 months for all frequencies tested (Figure 2K,L and Figure S3A–L; Table S4; *p* value between * *p* = 0.0451 and ** *p* = 0.0024). Previous work has demonstrated that suprathreshold wave I amplitude is closely associated with synapse preservation in IHCs [25,26]. These results therefore suggest that IHCs might be further affected by the loss of *Pex1 *gene.

### 3.3. VGlut3^Cre/Cre^Pex1^fl/fl^ Mice Exhibit Mild Hearing Loss and Preservation of Neural Central Gain

**Figure 3:**
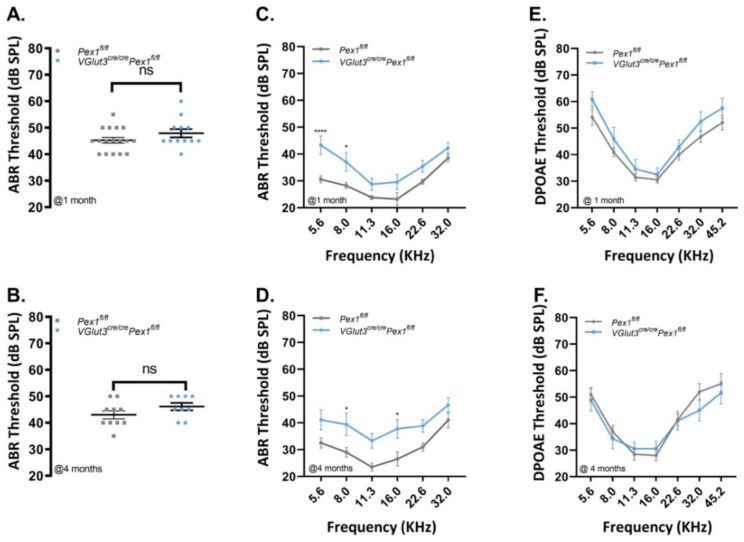
Mild hearing loss is observed in *VGlut3^cre/cre^Pex1^fl/fl^ mice*. (**A**,**B**) Click ABR recordings on *Pex1^fl/fl^* (grey) and *VGlut3^cre/cre^Pex1^fl/fl^* (blue) mice at 1 month (Table S5; mean ± S.E.M. ns *p* = 0.2243, Mann–Whitney test) (**A**) and 4 months (Table S5; mean ± S.E.M. ns *p* = 0.2962, Mann–Whitney test) (**B**); (**C**,**D**) Pure-tone ABR recordings as a function of frequencies (kHz) on *Pex1^fl/fl^* (grey) and *VGlut3^cre/cre^Pex1^fl/fl^* (blue) mice at 1 month (Table S5; mean ± S.E.M. 5.6 kHz: **** *p* = 0.00007, 8.0 kHz: * *p* = 0.01236, 11.3 kHz: ns *p* = 0.4983, 16.0 kHz: ns *p* = 0.1559, 22.6 kHz: ns *p* = 0.2691, 32.0 kHz: ns *p* > 0.999—2 way ANOVA Bonferroni’s multi-comparison test) (**C**) and 4 months (Table S5; mean ± S.E.M. 5.6 kHz: ns *p* = 0.1421, 8.0 kHz: * *p* = 0.0382, 11.3 kHz: ns *p* = 0.0604, 16.0 kHz: * *p* = 0.0199, 22.6 kHz: ns *p* = 0.2271, 32.0KHz: ns *p* = 0.8029—2way ANOVA Bonferroni’s multi-comparison test) (**D**); (**E**,**F**) Distortion-product otoacoustic emissions (DPOAEs) recordings as a function of frequencies (kHz) on *Pex1^fl/fl^* (grey) and *VGlut3^cre/cre^Pex1^fl/fl^* (blue) mice at 1 month (Table S6; mean ± S.E.M. 5.6 kHz: ns *p* = 0.5657, 8.0 kHz: ns *p* > 0.999, 11.3 kHz: ns *p* > 0.999, 16.0 kHz: ns *p* > 0.999, 22.6 kHz: ns *p* > 0.999, 32.0 kHz: ns *p* = 0.9486, 45.2 kHz: ns *p* > 0.999—2 way ANOVA Bonferroni’s multi-comparison test) (**E**) and 4 months (Table S6; mean ± S.E.M. 5.6 kHz: ns *p* > 0.999, 8.0 kHz: ns *p* > 0.999, 11.3 kHz: ns *p* > 0.999, 16.0 kHz: ns *p* > 0.999, 22.6 kHz: ns *p* > 0.999, 32.0 kHz: ns *p* = 0.8351, 45.2 kHz: ns *p* > 0.999—2 way ANOVA Bonferroni’s multi-comparison test) (**F**). 1 month: *Pex1^fl/fl^* (*n* = 17), *VGlut3^cre/cre^Pex1^fl/fl^* (*n* = 12), 4 months—*Pex1^fl/fl^* (*n* = 10), *VGlut3^cre/cre^Pex1^fl/fl^* (*n* = 9).

Work with the *Gfi1^Cre^* mouse line has previously revealed mosaic recombination in both IHCs and OHCs [27]. Furthermore, *Gfi1^Cre^* mice have been shown to induce recombination in other cell types within the inner ear [28] and display progressive hearing loss. Thus, to specifically investigate the function of *Pex1* in IHCs, we generated and analyzed auditory function of *VGlut3^Cre/Cre^Pex1^fl/fl^* mice. Similar to *Gfi1^Cre/+^Pex1^fl/fl^* mice, on click-evoked recording, we noticed similar ABR thresholds at 1 and 4 months of age for *VGlut3^Cre/Cre^Pex1^fl/fl^* mice compared to control *Pex1^fl/fl^* mice (at 1 month, *Pex1^fl/fl^: *45.29 ± 1.09 dB SPL, *n* = 17; *VGlut3^Cre/Cre^Pex1^fl/fl^*: 47.92 ± 1.56 dB SPL, *n* = 12—at 4 months, *Pex1^fl/fl^: *43.00 ± 1.53 dB SPL, *n* = 10; *VGlut3^Cre/Cre^Pex1^fl/fl^*: 46.11 ± 1.39 dB SPL, *n* = 10; Table S5; Figure 3A,B).

**Figure 4:**
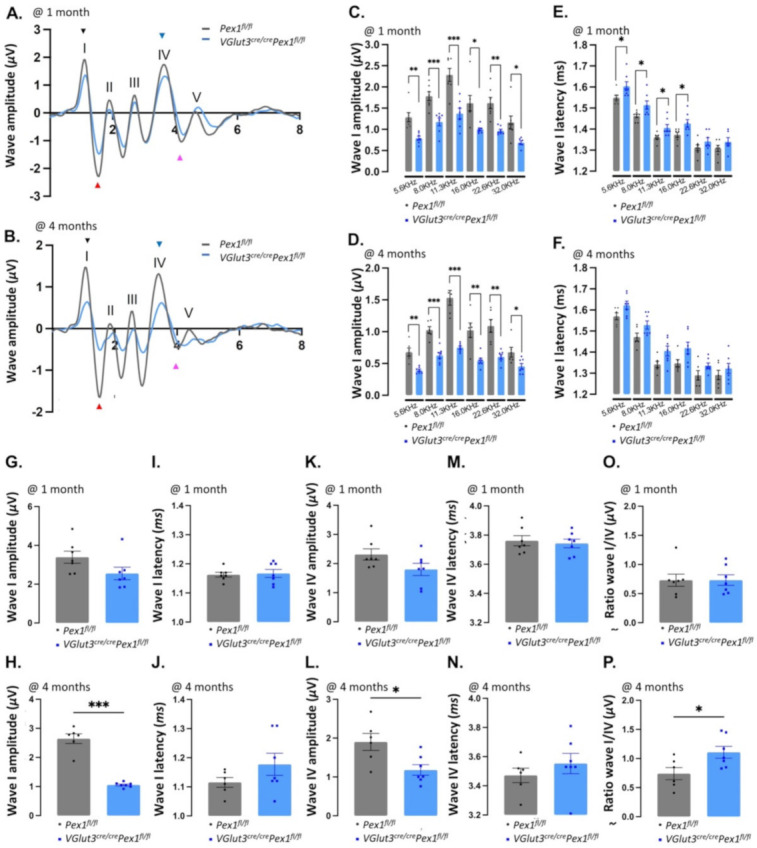
Wave I and IV amplitude are decreased *in VGlut3^cre/cre^Pex1^fl/fl^*. (**A**,**B**) Average of individual click ABR wave traces recorded on *Pex1^fl/fl^* (grey) and *VGlut3^cre/cre^Pex1^fl/fl^* (blue) mice at 1 month (**A**) and 4 months (**B**). Wave I, II, III, IV and V have been annotated. Black arrowhead indicates the positive peak of the wave I (P1), the red arrowhead indicates the negative peak of the wave I (N1), blue arrowhead indicate the positive peak of the wave IV (P4) and the pink arrowhead indicate the negative peak of the wave IV (N4). (**C**,**D**) Average of the wave I amplitude (N1-P1) on pure tones recorded from 5.6 to 32.0 kHz at 1 month (Table S7; mean ± S.E.M. 5.6 kHz: ** *p* = 0.0028, 8.0 kHz: *** *p* = 0.0009, 11.3 kHz: *** *p* = 0.0007, 16.0 kHz: * *p* = 0.0141, 22.6 kHz: ** *p* = 0.0022, 32.0 kHz: * *p* = 0.0213—unpaired *t*-test with Welch’s correction) (**C**) and 4 months (Table S7; mean ± S.E.M. 5.6 kHz: * *p* = 0.0048, 8.0 kHz: *** *p* = 0.0002, 11.3 kHz: *** *p* = 0.0009, 16.0 kHz: ** *p* = 0.0069, 22.6 kHz: ** *p* = 0.0036, 32.0 kHz: * *p* = 0.0368—unpaired *t*-test with Welch’s correction) (**D**) on *Pex1^fl/fl^* (grey) and *VGlut3^cre/cre^Pex1^fl/fl^* (blue) mice; (**E**,**F**) Average of the wave I latency on pure tones recorded from 5.6 to 32.0 kHz at 1 month (Table S8; mean ± S.E.M. 5.6 kHz: * *p* = 0.0210, 8.0 kHz: * *p* = 0.0390, 11.3 kHz: * *p* = 0.0175, 16.0 kHz: * *p* = 0.0146, 22.6 kHz: ns *p* = 0.2978, 32.0 kHz: ns *p* = 0.2477—Mann–Whitney test) (**E**) and 4 months (Table S8; mean ± S.E.M. 5.6 kHz: ns *p* = 0.0822, 8.0 kHz: ns *p* = 0.0629, 11.3 kHz: ns *p* = 0.0565, 16.0 kHz: ns *p* = 0.0542, 22.6 kHz: ns p = 0.1241, 32.0 kHz: ns *p* = 0.3910—Mann–Whitney test) (**F**) on *Pex1^fl/fl^* (grey) and *VGlut3^cre/cre^Pex1^fl/fl^* (blue) mice; (**G**,**H**) Average of wave I amplitude (N1-P1) on Click at 1 month (**G**) and 4 months (**H**) (Table S7; mean ± S.E.M. 1 month: ns *p* = 0.0842, 4 months: *** *p* = 0.0002—unpaired *t*-test with Welch’s correction) on *Pex1^fl/fl^* (grey) and *VGlut3^cre/cre^Pex1^fl/fl^* (blue) mice; (**I**,**J**) Average of the wave I latency on Click recording at 1 month (**I**) and 4 months (**J**) (Table S8; mean ± S.E.M.—1 month: ns *p* = 0.9825, 4 months: ns *p* = 0.3415—Mann–Whitney) on *Pex1^fl/fl^* (grey) and *VGlut3^cre/cre^Pex1^fl/fl^* (blue) mice; (**K**,**L**) Average of wave IV amplitude (N4-P4) on Click recording at 1 month (**K**) and 4 months (**L**) (Table S9; mean ± S.E.M.—1 month: ns *p* = 0.0943, 4 months: * *p* = 0.0217—unpaired *t*-test with Welch’s correction) on *Pex1^fl/fl^* (grey) and *VGlut3^cre/cre^Pex1^fl/fl^* (blue) mice; (**M**,**N**) Average of the wave IV latency on Click recording at 1 month (**M**) and 4 months (**N**) (Table S10; mean ± S.E.M. 1 month: ns *p* = 0.8269, 4 months: ns *p* = 0.1346—unpaired *t*-test with Welch’s correction) on *Pex1^fl/fl^* (grey) and *VGlut3^cre/cre^Pex1^fl/fl^* (blue) mice; (**O**,**P**) Ratio wave IV/I measured on Click recording at 1 month (**O**) and 4 months (**P**) (Table S11; mean ± S.E.M. -1 month: ns *p* = 0.9839, 4 months: * *p* = 0.0288—unpaired *t*-test with Welch’s correction) on *Pex1^fl/fl^* (grey) and *VGlut3^cre/cre^Pex1^fl/fl^* (blue) mice. Averages the highest intensity tested (90, 80, 70 dB SLP); 1 month: *Pex1^fl/fl^* (*n* = 7), *VGlut3^cre/cre^Pex1^fl/fl^* (*n* = 7), 4 months—*Pex1^fl/fl^* (*n* = 6), *VGlut3^cre/cre^Pex1^fl/fl^* (*n* = 7).

Interestingly, pure tones ABRs performed at frequencies ranging from 5.6 to 32.0 kHz show a progressive elevation of the threshold for all frequencies at both ages tested for *VGlut3^Cre/Cre^Pex1^fl/fl^* compared to control *Pex1^fl/fl^* mice (Table S5; Figure 3C,D; *p* value between * *p* = 0.0382 and **** *p* = 0.00007). While DPOAEs were not affected (Table S6; Figure 3E,F), representative ABR waveforms were altered with significant changes in the amplitude of the response, affecting all peaks (Figure 4A,B). We performed analysis of wave I amplitude and latency in response to pure tone stimuli, at 1 and 4 months in *VGlut3^Cre/Cre^Pex1^fl/fl^* compared to control *Pex1^fl/fl^* mice, averaging values for the three highest intensities tested (70, 80 and 90 dB SPL). This analysis shows that wave I amplitude is strongly reduced for all frequencies tested at 1 and 4 months of age (Table S7; Figure 4C,D; *p* value between * *p* = 0.0368 and *** *p* = 0.0002). Analysis of the wave I amplitude as a function of sound intensity also revealed significant change in slope values in most of the frequencies tested in *VGlut3^Cre/Cre^Pex1^fl/fl^* at 1 and 4 months (Figure S4A–L; Table S18; *p* value between * *p* = 0.0283 and *** *p* = 0.0007). Wave I latency was significantly altered in *VGlut3^Cre/Cre^Pex1^fl/fl^* mice at 1 month and slightly increased at 4 months for all frequencies tested (Table S8, Figure 4E,F and Figure S5A–L; *p* value between * *p* = 0.0390 and * *p* = 0.0146). Similarly, on click-evoked recording, the analysis of wave I in *VGlut3^Cre/Cre^Pex1^fl/fl^* mice revealed a progressive phenotype with a mild decrease (~25%) in ABR amplitude at 1 month of age (*Pex1^fl/fl^*: 3.39 ± 0.31, *n* = 7; *VGlut3^Cre/Cre^Pex1^fl/fl^*: 2.55 ± 0.32, *n* = 7; Table S7; Figure 4G) and a severe reduction (~60%) at 4 months (*Pex1^fl/fl^*: 2.65 ± 0.17, *n* = 6; *VGlut3^Cre/Cre^Pex1^fl/fl^*: 1.05 ± 0.03, *n* = 7; *** *p* = 0.0002; Table S7; Figure 4H). Additionally, we observed a progressive increase of the wave I latency over age of *VGlut3^Cre/Cre^Pex1^fl/fl^* mice compared to the control mice (1 month: *Pex1^fl/fl^*: 1.16 ± 0.01, *n* = 7; *VGlut3^Cre/Cre^Pex1^fl/fl^*: 1.17 ± 0.01, *n* = 7—4 months: *Pex1^fl/fl^*: 1.12 ± 0.02, *n* = 6; *VGlut3^Cre/Cre^Pex1^fl/fl^*: 1.18 ± 0.04, *n* = 7; Table S8; Figure 4I,J). These findings suggest that deletion of *Pex1* under *VGlut3 *promoter leads to mild hearing loss and has an impact on IHC function along the entire organ. Each ABR waveform is generated by the activation of anatomical sites along the auditory pathway in response to sound stimuli. While wave I corresponds to the activity of the distal part of the auditory nerve, wave IV is believed to mostly reflect a central component with activation of the superior olivary complex. To determine if the auditory abnormalities, previously noticed, were restricted to a dysfunction at the HC level, we analyzed wave IV amplitude and latency on click-evoked ABRs at 1 and 4 months of age in *VGlut3^Cre/Cre^Pex1^fl/fl^* mice. We found that wave IV amplitude was affected at both ages in *VGlut3^Cre/Cre^Pex1^fl/fl^* mice. However, the phenotype was more severe at 4 months in *VGlut3^Cre/Cre^Pex1^fl/fl^* mice with, respectively ~22% and ~38% wave IV amplitude decreased (at 1 month: *Pex1^fl/fl^*: 2.31 ± 0.19, *n* = 7; *VGlut3^Cre/Cre^Pex1^fl/fl^*: 1.80 ± 0.21, *n* = 7—at 4 months: *Pex1^fl/fl^*: 1.90 ± 0.22, *n* = 6; *VGlut3^Cre/Cre^Pex1^fl/fl^*: 1.18 ± 0.14, *n* = 7; * *p* = 0.0217; Table S9; Figure 4K,L). In contrast to 1 month, wave IV latency was slightly increased at 4 months of age in *VGlut3^Cre/Cre^Pex1^fl/fl^* mice (at 1 month: *Pex1^fl/fl^*: 3.76 ± 0.04, *n* = 7; *VGlut3^Cre/Cre^Pex1^fl/fl^*: 3.74 ± 0.03, *n* = 7—at 4 months: *Pex1^fl/fl^*: 3.47 ± 0.05, *n* = 6; *VGlut3^Cre/Cre^Pex1^fl/fl^*: 3.55 ± 0.07, *n* = 7; Table S10; Figure 4M,N). Central compensation for reduced cochlear input has previously been reported [29,30]. Compensation can be determined by analysis of wave IV/I ratio. To determine if compensation occurs in the *VGlut3^Cre/Cre^Pex1^fl/fl^* mice, we analyzed wave IV/I ratio at 1 and 4 months. At 1 month, a similar wave IV/I ratio was observed in *VGlut3^Cre/Cre^Pex1^fl/fl^* mice compared to control mice (*Pex1^fl/fl^*: 0.73 ± 0.10, *n* = 7; *VGlut3^Cre/Cre^Pex1^fl/fl^*: 0.73 ± 0.09, *n* = 7 -Table S11, Figure 4O), meaning that no neural gain was observed at this stage, perhaps due to the limited reduction in wave I reported at 1 month (~25%). However, by 4 months of age, a significant increase (~50%, * *p* = 0.0288) of wave IV/I ratio was observed in *VGlut3^Cre/Cre^Pex1^fl/fl^* compared to control *Pex1^fl/f^* mice (*Pex1^fl/fl^*: 0.74 ± 0.10, *n* = 6; *VGlut3^Cre/Cre^Pex1^fl/fl^*: 1.11 ± 0.10, *n* = 7—Table S11, Figure 4P). Data show that *VGlut3^Cre/Cre^Pex1^fl/fl^* mice presented a disproportionally central response (larger wave IV amplitude) relative to the decreased wave I amplitude, ~60% at this age. Collectively, these data show that *VGlut3^Cre/Cre^Pex1^fl/fl^* mice compensate centrally for the reduced cochlear input by generating central neural gain. This result suggests that negative impacts of the deletion of *Pex1* under the *VGlut3* promoter mainly occurs at the IHCs level.

### 3.4. Synaptopathy Is Observed in VGlut3^Cre/Cre^Pex1^fl/fl^

**Figure 5:**
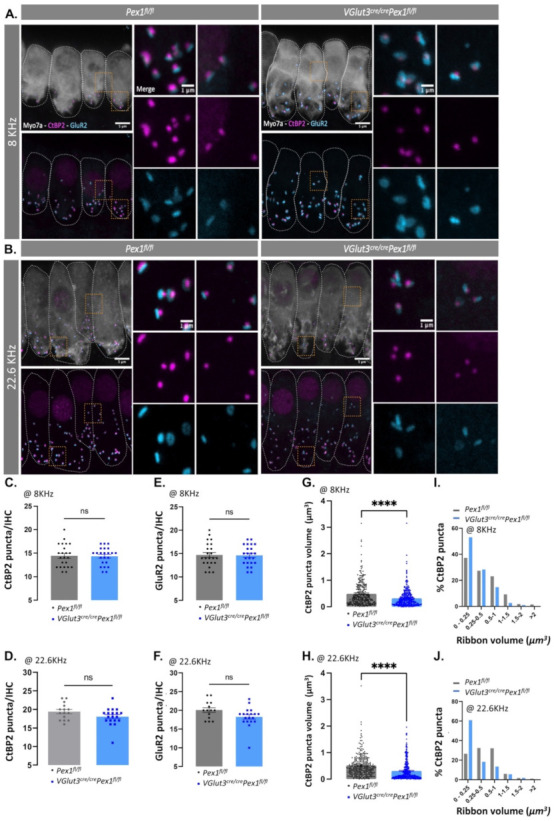
*Pex1* defect leads to smaller ribbon synapses. (**A**,**B**) Confocal images of inner hair cells (IHCs) at 8.0 (**A**) and 22.6 kHz (**B**) region in *Pex1^fl/fl^* and *VGlut3^cre/cre^Pex1^fl/fl^* mice stained with a CtBP2 (magenta—pre-synaptic), GluR2 (cyan—post-synaptic) and Myo7a (grey—IHCs). Scale bar: 5 and 1 µm. Orange square highlight the zoom in region in the right panel; (**C**,**D**) Quantification of CtBP2 puncta per IHC at 8.0 kHz (Table S12; Mean ± S.E.M. ns *p* = 0.9513, *Pex1^fl/fl^* (mice: *n* = 6—IHC: *n* = 23), *VGlut3^cre/cre^Pex1^fl/fl^* (mice: *n* = 6—IHC: *n* = 23)) **(C)** and 22.6 kHz (Table S12; Mean ± S.E.M. ns *p* = 0.0796, *Pex1^fl/fl^* (mice: *n* = 5—IHC: *n* = 15), *VGlut3^cre/cre^Pex1^fl/fl^* (mice: *n* = 5—IHC: *n* = 19)) (**D**); (**E**,**F**) Quantification of GluR2 puncta per IHC at 8.0 kHz (Table S1; Mean ± S.E.M. ns *p* = 0.9866, *Pex1^fl/fl^* (mice: *n* = 6—IHC: *n* = 23), *VGlut3^cre/cre^Pex1^fl/fl^* (mice: *n* = 6—IHC: *n* = 23)) (**E**) and 22.6 kHz (Table S12; Mean ± S.E.M. ns *p* = 0.0524, *Pex1^fl/fl^* (mice: *n* = 4—IHC: *n* = 14), *VGlut3^cre/cre^Pex1^fl/fl^* (mice: *n* = 5—IHC: *n* = 19)) (**F**) region; (**G**,**H**) Volume of CtBP2 puncta at 8.0 kHz (Table S13; Mean ± S.E.M. **** *p* < 0.0001, *Pex1^fl/fl^* (mice: *n* = 6—ribbon: *n* = 282), *VGlut3^cre/cre^Pex1^fl/fl^* (mice: *n* = 6—ribbon: *n* = 311)) (**G**) and 22.6 kHz (Table S13; Mean ± S.E.M. **** *p* < 0.0001, *Pex1^fl/fl^* (mice: *n* = 5—ribbon: *n* = 286), *VGlut3^cre/cre^Pex1^fl/fl^* (mice: *n* = 5—ribbon: *n* = 328)) (**H**); (**I**,**J**) CtBP2 puncta (%) as a function of ribbon volume (μm^3^) at 8.0 (**I**) and 22.6 kHz (**J**) (Table S13).

We hypothesized that the decrease in wave I amplitude in the *VGlut3^Cre/Cre^Pex1^fl/fl^* resulted from defects in the synapse. To investigate whether the deletion of *Pex1* under the *VGlut3* promoter was affecting the synaptic region, we first examined the number of ribbons in IHCs of control and mutant mice. To that end, we labeled the pre-synaptic ribbon using anti-CtBP2 antibody and the post-synaptic ribbon using anti-GluR2 antibody. We labelled IHCs body using anti-Myo7a antibody and the cuticular plate using phalloidin toxins (Figure 5A,B). At 4 months of age, we analyzed two regions of the organ: 8.0 and 22.6 kHz. No obvious changes in the number of CtBP2 puncta were observed in *VGlut3^Cre/Cre^Pex1^fl/fl^* compared to control *Pex1^fl/fl^* mice (at 8.0 kHz: *Pex1^fl/fl^*: 14.43 ± 0.51, *n* = 23 IHCs; *VGlut3^Cre/Cre^Pex1^fl/fl^*: 14.35 ± 0.39, *n* = 23 IHCs—at 22.6 kHz: *Pex1^fl/fl^*: 19.81 ± 0.67, *n* = 15 IHCs; *VGlut3^Cre/Cre^Pex1^fl/fl^*: 18.05 ± 0.55, *n* = 19 IHCs; Table S12; Figure 5C,D). Similarly, no significantly changes were noticed on GluR2 puncta quantification between *VGlut3^Cre/Cre^Pex1^fl/fl^* and control *Pex1^fl/fl^* mice (at 8.0 kHz: *Pex1^fl/fl^*: 14.70 ± 0.54, *n* = 23 IHCs; *VGlut3^Cre/Cre^Pex1^fl/fl^*: 14.61 ± 0.45, *n* = 23 IHCs—at 22.6 kHz: *Pex1^fl/fl^*: 20.07 ± 0.63, *n* = 14 IHCs; *VGlut3^Cre/Cre^Pex1^fl/fl^*: 18.26 ± 0.61, *n* = 19 IHCs; Table S12; Figure 5E,F). In addition, the same percentage of paired ribbon synapses, defined by co-localization of CtBP2 and GluR2 staining, were observed in control and mutant mice, meaning that there were no orphan ribbons present (Table S19; Figure S6A). To further evaluate the distribution of ribbons synapses along the pillar/modiolar axis of the IHCs, we performed a *k*-means clustering function analysis of the images acquired. This analysis revealed similar organization in *VGlut3^Cre/Cre^Pex1^fl/fl^* mice as in *Pex1^fl/fl^* control mice at 8.0 kHz (Pillar: *Pex1^fl/fl^*: 45.57 ± 2.98, *n* = 20 IHCs; *VGlut3^Cre/Cre^Pex1^fl/fl^*: 38.96 ± 5.33, *n* = 15 IHCs—Modiolar: *Pex1^fl/fl^*: 54.43 ± 3.0, *n* = 20 IHCs; *VGlut3^Cre/Cre^Pex1^fl/fl^*: 61.05 ± 5.33, *n* = 15 IHCs; Table S19; Figure S6B), and 22.6 kHz (Pillar: *Pex1^fl/fl^*: 46.63 ± 3.65, *n* = 15 IHCs; *VGlut3^Cre/Cre^Pex1^fl/fl^*: 44.10 ± 3.65, *n* = 12 IHCs—Modiolar: *Pex1^fl/fl^*: 53.37 ± 3.03, *n* = 15 IHCs; *VGlut3^Cre/Cre^Pex1^fl/fl^*: 55.91 ± 3.65, *n* = 12 IHCs; Table S19; Figure S6C). Nevertheless, changes in pre-synaptic ribbon volume were observed. To quantify ribbon volume, we measured the volume of CtBP2 puncta at 4 months of age at 8.0 and 22.6 kHz regions in *VGlut3^Cre/Cre^Pex1^fl/fl^* and control *Pex1^fl/fl^* mice using Imaris software. A significant decrease (*p* < 0.0001) in ribbon volume was observed in *VGlut3^Cre/Cre^Pex1^fl/fl^* compared to control *Pex1^fl/fl^* mice in both the 8.0 and 22.6 kHz regions, respectively, with ~35% and ~40% decrease (at 8.0 kHz: *Pex1^fl/fl^*: 0.49 ± 0.03, *n* = 282 ribbons; *VGlut3^Cre/Cre^Pex1^fl/fl^*: 0.32 ± 0.02, *n* = 311 ribbons—at 22.6 kHz: *Pex1^fl/fl^*: 0.52 ± 0.02, *n* = 286 ribbons; *VGlut3^Cre/Cre^Pex1^fl/fl^*: 0.31 ± 0.02, *n* = 328 ribbons; Table S13; Figure 5G,H).

Accordingly, an increase in smaller ribbon synapses (from 0 to 0.25 μm^3^) was evident at both 8.0 and 22.6 kHz, respectively with ~16% and ~35% increase in *VGlut3^Cre/Cre^Pex1^fl/fl^* compared to control *Pex1^fl/fl^* mice (at 8.0 kHz: *Pex1^fl/fl^*: 37.23 %, *n* = 282 ribbons; *VGlut3^Cre/Cre^Pex1^fl/fl^*: 53.05%, *n* = 311 ribbons—at 22.6 kHz: *Pex1^fl/fl^*: 26.57 %, *n* = 286 ribbons; *VGlut3^Cre/Cre^Pex1^fl/fl^*: 60.98, *n* = 328 ribbons; Table S13; Figure 5I,J).

Taken together, these results demonstrate that *Pex1* disruption in HCs leads to disruption of ribbon synapses and alteration in transmission of the sensory signal at the distal end of the auditory nerve.

### 3.5. IHCs from VGlut3^Cre/Cre^Pex1^fl/fl^ Mice Present Altered Exocystosis

**Figure 6:**
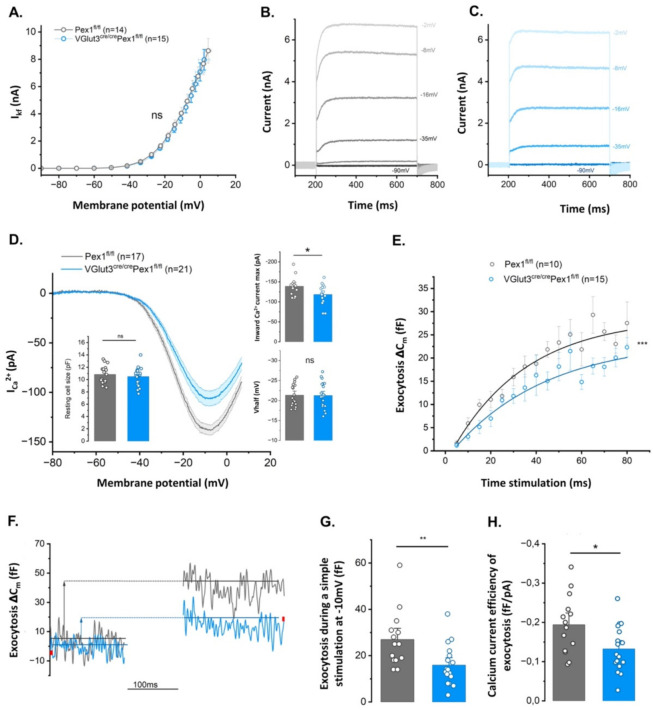
Lack of Pex1 in P18–P21 IHCs affects Ca^2+^ currents and exocytosis while no alteration in BK channels is observed. (**A**) Comparative BK current-voltage curve (Table S14, *n* = 14 and 15, respectively in P18–P21 *Pex1^fl/fl^* and *VGlut3^cre/cre^Pex1^fl/fl^*; ns *p* = 0.65). IV curve is obtained with 20 ms depolarizing steps from −90 mV to +70 mV with an increment of +10 mV for each depolarizing step with IK,f measured at 2 ms from the onset of the depolarizing step; (**B**,**C**) Potassium current responses from *Pex1^fl/fl^* IHCs (grey) and *VGlut3^cre/cre^Pex1^fl/fl^* (blue). Currents were elicited using 500 ms depolarizing steps from −90 mV to +70 mV with an increment of +10 mV for each depolarizing step; (**D**) Comparative Ca^2+^ current-voltage curve (Table S14, IV; *n* = 17 and 21, respectively for P21–P25 *Pex1^fl/fl^* in grey and *VGlut3^cre/cre^Pex1^fl/fl^* in blue). IV curve is obtained with a ramp protocol starting at −80 mV to 10 mV with an increase of 1 mV each millisecond. Values in the right graphs indicate the max inward Ca^2+^ current and the V1/2 of the IV curve. The peak of the Ca^2+^ current was significantly higher in *Pex1^fl/fl^* IHCs compared to *VGlut3^cre/cre^Pex1^fl/fl^* IHCs (Table S14; unpaired *t*-test, * *p* = 0.025). While the Ca^2+^ current is decreased there is no shift in the activation of the Ca^2+^ channels of *VGlut3^cre/cre^Pex1^fl/fl^* IHCs (Table S14; unpaired *t*-test, ns *p* = 0.93). Insert graph in left: comparative mean IHC resting membrane capacitance, measured at −70 mV, in whole-cell patch clamp configuration (Table S14; *n* = 17 and 21, respectively in *Pex1^fl/fl^* and *VGlut3^cre/cre^Pex1^fl/fl^*; unpaired *t*-test, ns *p* = 0.47); (**E**) Comparative kinetics of exocytosis show a significant decrease of the fast exocytosis of the Readily Releasable Pool of vesicles in *VGlut3^cre/cre^Pex1^fl/fl^* IHCs (2way ANOVA, *** *p* = 7.6 × 10^−8^). Exocytosis was elicited with voltage steps from −80 mV to −10 mV (corresponding to the max inward Ca^2+^ current) with increasing duration from 5 ms to 80 ms. (**F**) Representative examples of a simple sustained exocytosis recordings (ΔC_m_) in *Pex1^fl/fl^* and *VGlut3^cre/cre^Pex1^fl/fl^* IHCs during a 100 ms voltage step stimulation from −80 mV to −10 mV; (**G**) Values in graph show a significant decrease of exocytosis during a sustained (100 ms) voltage step stimulation from −80 mV to −10 mV in *VGlut3^cre/cre^Pex1^fl/fl^* IHCs (Table S14; *n* = 14 and 17, respectively in *Pex1^fl/fl^* and *VGlut3^cre/cre^Pex1^fl/fl^*; unpaired *t*-test, ** *p* = 0.006); (**H**) Exocytosis Ca^2+^ efficiency is also decreased in *VGlut3^cre/cre^Pex1^fl/fl^* IHCs showing that the decreased in exocytosis is not only due to a decrease in calcium current (Table S14; *n* = 14 and 17, respectively in *Pex1^fl/fl^* and *VGlut3^cre/cre^Pex1^fl/fl^*; unpaired *t*-test, * *p* = 0.015).

BK (K Ca1.1) channels are known to be highly sensitive to oxidative stress. Changes in fast-repolarizing BK channels have been associated with aging and disruptions in the hair cell synapse [19]. To determine if *Pex1* disruption affects the functional maturation of IHCs in *VGlut3^Cre/Cre^Pex1^fl/fl^* mice, we recorded K^+^ voltage dependent currents in P18-P21 mature IHCs from *Pex1^fl/fl^* and *VGlut3^Cre/Cre^Pex1^fl/fl^* mice. No significant changes in conductance, kinetic and voltage-dependent activation of the K^+^ currents were observed in *VGlut3^Cre/Cre^Pex1^fl/fl^* mice (Figure 6A–C; Table S14; two-way ANOVA, ns *p* = 0.65). We also assessed membrane capacitance at rest to determine if changes in cell sizes were associated with the conditional deletion of *Pex1* and we observed no significant changes (Figure 6D; Table S14; *Pex1^fl/fl^*: 10.82 ± 0.35 pF—*VGlut3^cre/cre^Pex1^fl/fl^*:10.48 ± 0.31 pF; unpaired *t*-test, ns *p* = 0.47).

Next, we investigated changes in exocytosis and Ca^2+^ current in mature P21-P25 IHCs from *Pex1 ^fl/fl^* and *VGlut3^Cre/Cre^Pex1 ^fl/fl^* mice to determine if *Pex1* disruption affects ribbon synapse function. Ca^2+^ currents are crucial to trigger fusion of the synaptic vesicles in the IHC synaptic active zone. Depolarization of IHC activates Ca_V_1.3 Ca^2+^ channels. These channels are present in the synaptic active zone, forming tight clusters with ribbons that aggregate synaptic vesicles [31,32]. Whole-cell patch clamp recordings of IHCs demonstrate significant decrease of inward Ca^2+^ currents in *VGlut3^Cre/Cre^Pex1^fl/fl^* mice (Figure 6D; Table S14; *Pex1^fl/fl^*: 138.8 ± 6.3 pA—*VGlut3^cre/cre^Pex1^fl/fl^*: −118.4 ± 5.89 pA; unpaired *t*-test, * *p* = 0.025), with, however, no shift in the activation curve (Figure 6D; Table S14; *Pex1^fl/fl^*: 21.36 ± 0.67 mV—*VGlut3^cre/cre^Pex1^fl/fl^*: −21.27 ± 0.68 mV; unpaired *t*-test, ns *p* = 0.93).

Exocytosis is elicited by the fusion of the synaptic vesicles to the cell membrane followed by the released of the neurotransmitter in the synaptic cleft. The ribbon structure permits synaptic vesicles aggregation to the IHC active zone that are ready to fuse at the membrane called Readily Releasable Pool of vesicles (RRP). RRP vesicles are crucial for the temporal precision of a fast exocytosis [33,34]. Aggregation of multiple vesicles by the ribbon facilitates vesicles recruitment and permit a sustained exocytosis [35]. Kinetics of brief stimulations (from 5 to 80 ms) addressing the released of RRP vesicles was impaired by *Pex1 *disruption (Figure 6E; Table S14; two-way ANOVA, *** *p* = 7.6 × 10^−8^). Sustained exocytosis during a 100 ms voltage-step stimulation from −80 mV to −10 mV was also significantly reduced in IHC lacking *Pex1* (Table S14, Figure 6F,G; *Pex1^fl/fl^*: 26.93 ± 3.25 fF—*VGlut3^cre/cre^Pex1^fl/fl^*: 15.93 ± 2.11 fF; unpaired *t*-test, ** *p* = 0.006).

Since Ca^2+^ influx and exocytosis are tightly coupled, reduced exocytosis may result from the decrease in Ca^2+^ currents. However, we also noticed a significant decrease in the Ca^2+^ efficiency of exocytosis in IHCs of *VGlut3^Cre/Cre^Pex1^fl/fl^* mice (Figure 6H; Table S14; *Pex1^fl/fl^*: −0.194 ± 0.02 fF/pA—*VGlut3^cre/cre^Pex1^fl/fl^*: −0.132 ± 0.014 fF/pA; unpaired *t*-test, * *p* = 0.015). These results demonstrate that *Pex1* disruption leads to a defect in Ca^2+^ influx, a defect in fast and sustained exocytosis and is also paired with a defect in the coupling between Ca_V_1.3 channels and synaptic vesicles.

### 3.6. Pex1 Deletion Affects Peroxisomal Number

**Figure 7:**
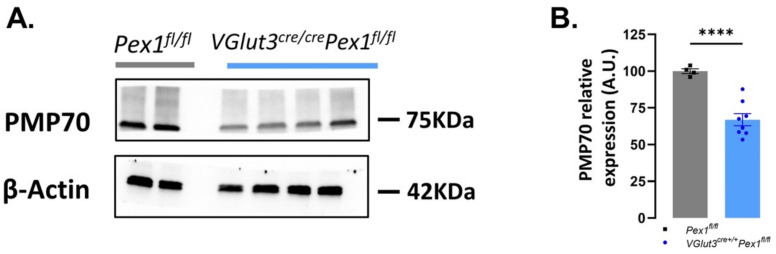
Decrease of relative expression of PMP70 in *VGlut3^cre/cre^Pex1^fl/fl^* mice. (**A**) Western blot performed on *Pex1^fl/fl^* and *VGlut3^cre/cre^Pex1^fl/fl^* mice at 1.5 months of age on liver tissue; (**B**) Quantification of the relative expression of PMP70 protein (Table S15—Mean ± S.E.M. **** *p* < 0.0001, unpaired *t*-test with Welch’s test; *Pex1^fl/fl^: n* = 4, *VGlut3^cre/cre^Pex1^fl/fl^*: *n* = 8).

Peroxin proteins are indispensable for peroxisomal biogenesis. At the same time, alterations in peroxins involved in the import of peroxisomal matrix proteins (PMP), such as Pex1, has been shown to lead to pexophagy [36,37]. We hypothesize that peroxisomal number is severally affected in absence of Pex1 protein. To assess peroxisomal number, we performed western blot analysis of PMP70 expression in *VGlut3^Cre/Cre^Pex1^fl/fl^* liver tissue, a tissue rich in peroxisomes also expressing *VGlut3*. PMP70 is a major component of peroxisomal membranes and disruptions in PMP70 can pinpoint to aberrant peroxisomal assembly. Here, we show significant decrease (~33%, **** *p* < 0.0001) of PMP70 protein (peroxisomal membrane protein) at 1.5 months of age in *VGlut3^Cre/Cre^Pex1^fl/fl^* compared to control mice (*Pex1^fl/fl^*: *n* = 4; *VGlut3^Cre/Cre^Pex1^fl/fl^*: *n* = 8; Table S15; Figure 7A,B).

This result confirms that loss of Pex1 protein affects the stability of peroxisomes. A similar phenotype is likely recapitulated in HCs of the inner ear. Unfortunately, specific changes, in IHCs only, could not be determined by global protein assessment of inner ear tissues from *VGlut3^Cre/Cre^Pex1^fl/fl^*, likely due to the low number of hair cells over the total cell population expressing peroxisomal proteins in our samples.

## 4. Discussion

In this study we demonstrate the essential role of Pex1 protein in inner hair cells. While progressive sensory-neural hearing loss has been reported to be associated with PBD-ZSD [3], the etiology of the disease, in the ear, has remained unknown. Mutations in *PEX1* gene are more commonly associated with PBD-ZSD. While prior studies have highlighted the function of *Pex1* in the retina and liver [38,39,40], no studies have explored how disruption in peroxisomes affects the auditory system and leads to hearing dysfunction [37]. As *Pex1 *is ubiquitously expressed, total deletion of this gene is neonatal-lethal in mice, thereby preventing functional studies in young and adult animals. To overcome this limitation, we sought to develop two new conditional *Pex1* mouse models. Given the high similarity of murine and human *Pex1* genes (~80%), we hypothesized that early deletion of *Pex1,* specifically in inner ear tissue, would lead to progressive hearing loss as reported in *PEX1* patients. We used two mouse models with deletions of *Pex1* specifically in hair cells, *Gfi1^Cre/^*^+^*Pex1^fl/fl^* (both IHCs and OHCs) and *VGlut3^Cre/Cre^Pex1^fl/fl^* (IHCs only). While DPOAEs were not altered, analysis of ABR thresholds and waveforms revealed changes in ABR thresholds from one to four months in *VGlut3^Cre/Cre^Pex1^fl/fl^*, and ABR wave I in both models. Our results demonstrate that IHCs are especially vulnerable to disruption on *Pex1* expression. IHC are the primary sensory receptors of the auditory organ. These cells have been shown to be especially vulnerable to noise exposure and aging [25,41,42,43]. Cochlear damage associated with noise and aging, however, typically starts with alterations in the IHC synapse with no change in ABR threshold but decrease in wave I amplitude [44]. Wave I is generated by the distal portion of the auditory nerve at the IHCs synapse. Decreases in wave I amplitude have been associated with defects in the synapse, so called synaptopathy. Here, we show that, while the number of ribbon synapses was unchanged, their volume was severely affected with a worsened phenotype in *VGlut3^Cre/Cre^Pex1^fl/fl^*. A similar result has been previously documented in a rodent model after noise exposure [45]. Electrophysiological recordings from IHCs of conditional KO mice also revealed functional alteration of the synapse with reduced exocytosis. Such phenotype, if also present in *PEX1* patients, would predict high sensitivity to noise induced hearing loss along with progressive hearing loss. Progression while mild, was indeed observed in *PEX1* patients [3].

Peroxisomes are small ubiquitous organelles involved in a variety of metabolic reactions, such as lipid biogenesis and reactive oxygen species (ROS) products detoxification. Previous work has demonstrated the essential function of peroxisomes against noise exposure, specifically through Pejvakin-mediated pexophagy (selective degradation of damaged peroxisomes) which protects auditory hair cells from oxidative stress [46]. Moreover, a previous study has shown that exposure to loud sounds leads to peroxisomal proliferation in cochlear hair cells [47]. This proliferation has been hypothesized to be a physiologically protecting response associated with increase in ROS exacerbated by loud sound exposure [47]. As peroxisomal proliferation cannot occur in absence of Pex1 protein, we suspect that this protective mechanism is severely impacted in *Pex1* mutant mice. The balance between peroxisome biogenesis and degradation is crucial for redox cell homeostasis. Our work shows that IHCs and IHC/Spiral ganglion synapses are particularly vulnerable to alteration in Pex1 protein expression and peroxisomal biogenesis. As hair cells do not regenerate, protective mechanisms are crucial for their survival. As such, we predict that *Pex1* mutant mice will display additional vulnerability to damaging sound exposure and aging.

Mechanistically, the loss of Pex1 protein is likely to impair recycling of Pex5 receptor, indispensable for the import of antioxidant enzymes, such as catalase. As such, Pex1 impairment is expected to lead to rapid accumulation of ROS products leading to activation of the pexophagy pathway [8,48,49]. Interestingly, 65% of PDB-ZSD phenotypes are associated with pexophagy [36]. As such, we propose that hearing loss in *PEX1* patients is the result of oxidative stress imbalance, increase in pexophagy and, at least initially, IHCs dysfunction.

Some caveats in this study must be noted. The phenotype observed in the conditional mice, used for our study, does not completely phenocopy the hearing phenotype observed in *PEX1* patients. This may be due to incomplete deletion of *Pex1* gene as even low expression levels of Pex1 protein (~5–15%) can prevent severe forms of PBD-ZSD [50]. In our study, ~15% of the full-length Pex1 protein remains in liver tissues which suggests incomplete penetrance of the *Cre *excision. It should also be noted that while previous studies have demonstrated strong expression of *VGlut3* in IHCs, *VGlut3* is also expressed in other cell types, including OHCs, albeit at a lower level [51,52,53]. Accordingly, we observed loss of OHCs in 4 months old *VGlut3^Cre/Cre^Pex1^fl/fl^* (data not shown). Furthermore, as peroxisomes are ubiquitous, we cannot exclude the role of other cell types/regions in the disease phenotype. The stria vascularis is a highly metabolic region of the inner ear that plays a role in generation of the endolymphatic potential and oxidative metabolism [54,55]. Intermediate cells of the stria vascularis derived from neural crest melanocytes are rich in peroxisomes [56] and largely implicated in the generation of the endocochlear potential. Disruption in stria function would have dire consequences to the function and survival of the sensory cells and would lead to severe hearing loss. Similarly, disruption in spiral ganglion neurons would also lead to auditory neuropathy and hearing loss.

In addition, a recent study investigating the role of another *Pex* gene in the ear, *Pex3* (*Pex3^tm1a^* mutant mice), similarly demonstrates mild progressive hearing loss in the high frequency range that is associated with a decrease in ABR wave I amplitude and synaptopathy with presence of orphan ribbon synapses and no change in ribbon number [57]. However, *Sox10 Cre* excision (targeting all inner ear cell type), in *Pex3^tm1d^* mice, lead to worsened hearing phenotype across all frequencies, decrease of the ABR wave I amplitude along with synaptic defects that included presence of orphan ribbons and decrease of ribbon number. These results highlight the role of *Pex3* in the inner ear and reinforce the notion that *Pex1*, as well as *Pex3* genes, are indispensable for the maintenance and proper function of sensory hair cells of the inner ear.

In summary, this study demonstrates the essential role of *Pex1* in HC development and function. The use of conditional *Pex1* mouse models can be further explored in other cell types in the auditory organ and can also be used to explore pathophysiology associated with *Pex1 *disruption in other organs such as liver, kidney, or brain.

## Figures and Tables

**Table 1:** Primer sets used for genotyping.

		Primer Sequence	Product
*** Pex1^fl/fl^***	Forward	5′-GAAGCATCCCTGCTCACTTC-3′	WT = 249 bp Floxed = 430 bp
	Reverse	5′-CCCTTCCACATACTAGGCAAGC-3′	
*** Gfi1^Cre^***	* Cre *	5′-GCCCAAATGTTGCTGGATAGT-3′	WT = 600 bp *Cre* = 700 bp
	Forward	5′-GGGATAACGGACCAGTTG-3′	
	Reverse	5′-CCGAGGGGCGTTAGGATA-3′	
*** VGlut3^Cre^***	* Cre *	5′-ATCGACCGGTAATGCAGGCAA-3′	WT = 300 bp *Cre* = 800 bp
	Forward	5′-GATGTCTTATGGAGCCACCACCCAG-3′	
	Reverse	5′-CGTAGACCAAGGTCCATATTCCCA-3′	

**Table 2:** Resources list.

Software	Source	More Information
Imaris Cell Imaging 9.6.1	Oxford Instruments (Abingdon, UK)	https://imaris.oxinst.com/ (accessed on 1 November 2022)
ImageJ- Fiji	NIH (Bethesda, MD, USA)	https://imagej.nih.gov/ij/index.html (accessed on 1 November 2022)
GraphPad Prism 9.1	California USA	https://www.graphpad.com/ (accessed on 1 November 2022)
OriginPro 2022b	Northampton, MA, USA	https://www.originlab.com/2022 (accessed on 1 November 2022)
ZEN 2.3 (blue edition)	Carl Zeiss (Jena, Germany)	https://www.zeiss.com/microscopy/en/products/software/zeiss-zen.html#zenversions (accessed on 1 November 2022)
EPL Cochlear Function Test Suite	EPL Engineering, Boston, MA, USA	https://www.masseyeandear.org/research/otolaryngology/eaton-peabody-laboratories/engineering-core (accessed on 1 November 2022)

## Data Availability

Data are all included with the supplemental material. Original data are available upon request.

## Supplementary Materials

The following supporting information can be downloaded at: https://www.mdpi.com/article/10.3390/cells11243982/s1. Figure S1: Characterization of new organ specific *Pex1* knockout mice; Figure S2: Decrease of the wave I amplitude observed over age during the recording of auditory brainstem responses (ABRs) in *Gfi1^cre/+^Pex1^fl/fl^* mice compared to control (*Pex1^fl/fl^*); Figure S3: Slight increase of the wave I latency measured on ABR recordings in *Gfi1^cre/+^Pex1^fl/fl^* mice compared to control (*Pex1^fl/fl^*); Figure S4: Decrease of the wave I amplitude observed over age during ABR recordings in *VGlut3^cre/cre^Pex1^fl/fl^* mice compared to control (*Pex1^fl/fl^*); Figure S5: Slight elevation of the wave I latency measured on ABR recordings in *VGlut3^cre/cre^Pex1^fl/fl^* mice compared to control (*Pex1^fl/fl^*); Figure S6: Unchanged distribution of ribbon synapses along Modiolar/Pillar axis in *VGlut3^cre/cre^Pex1^fl/fl^* compared to control mice (*Pex1^fl/fl^*). Table S1 to S19 are also included.
