# Supplementary material for: Loss of Pex1 in Inner Ear Hair Cells Contributes to Cochlear Synaptopathy and Hearing Loss
Source: Cells. 2022 Dec 9;11(24):3982. doi: 10.3390/cells11243982 (PMC9777190; doi:10.3390/cells11243982)
Supplement: Supplementary file 1 [file cells-11-03982-s001.zip › cells-2062987-supplementary.pdf]

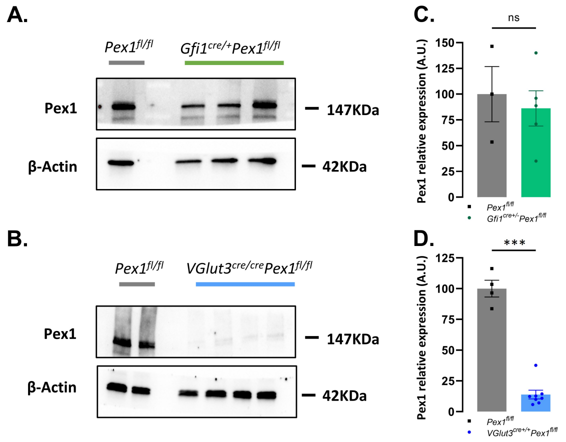

A.
Pex1fl/fl
Gfi1cre/+Pex1fl/fl
Pex1
147KDa
β-Actin
42KDa
B.
Pex1fl/fl
VGlut3cre/crePex1fl/fl
Pex1
147KDa
β-Actin
42KDa
C.
ns
Pex1 relative expression (A.U.)
150
125
100
75
50
25
0
Pex1fl/fl
Gfi1cre+/-Pex1fl/fl
D.
***
Pex1 relative expression (A.U.)
125
100
75
50
25
0
Pex1fl/fl
VGlut3cre+/+Pex1fl/fl

Figure S1: Characterization of new organ specific *Pex1* knockout mice.
(A-B) Western blot analysis of Pex1 protein expressed in liver at 6 weeks of age from *Gfi1*$^{cre/+}$*Pex1*$^{fl/fl}$ (table S16; Pex1$^{fl/fl}$ (*n* = 3), *Gfi1*$^{cre/+}$*Pex1*$^{fl/fl}$ (*n* = 5)); **(B)** and *VGlut3*$^{cre/cre}$*Pex1*$^{fl/fl}$ mice line (*Pex1*$^{fl/fl}$ (*n* = 4), *Gfi1*$^{cre/+}$*Pex1*$^{fl/fl}$ (*n* = 8)); (**C-D**) Quantification of Pex1 protein expression normalized to β-actin in *Gfi1*$^{cre/+}$*Pex1*$^{fl/fl}$ (table S16 – Mean ± S.E.M. - ns *p* = 0.6893, unpaired t-test with Welch's correction) **(C)** and *VGlut3*$^{cre/cre}$*Pex1*$^{fl/fl}$ (table S16 – *** *p* = 0.0001, unpaired t-test with Welch's correction) **(D)**.

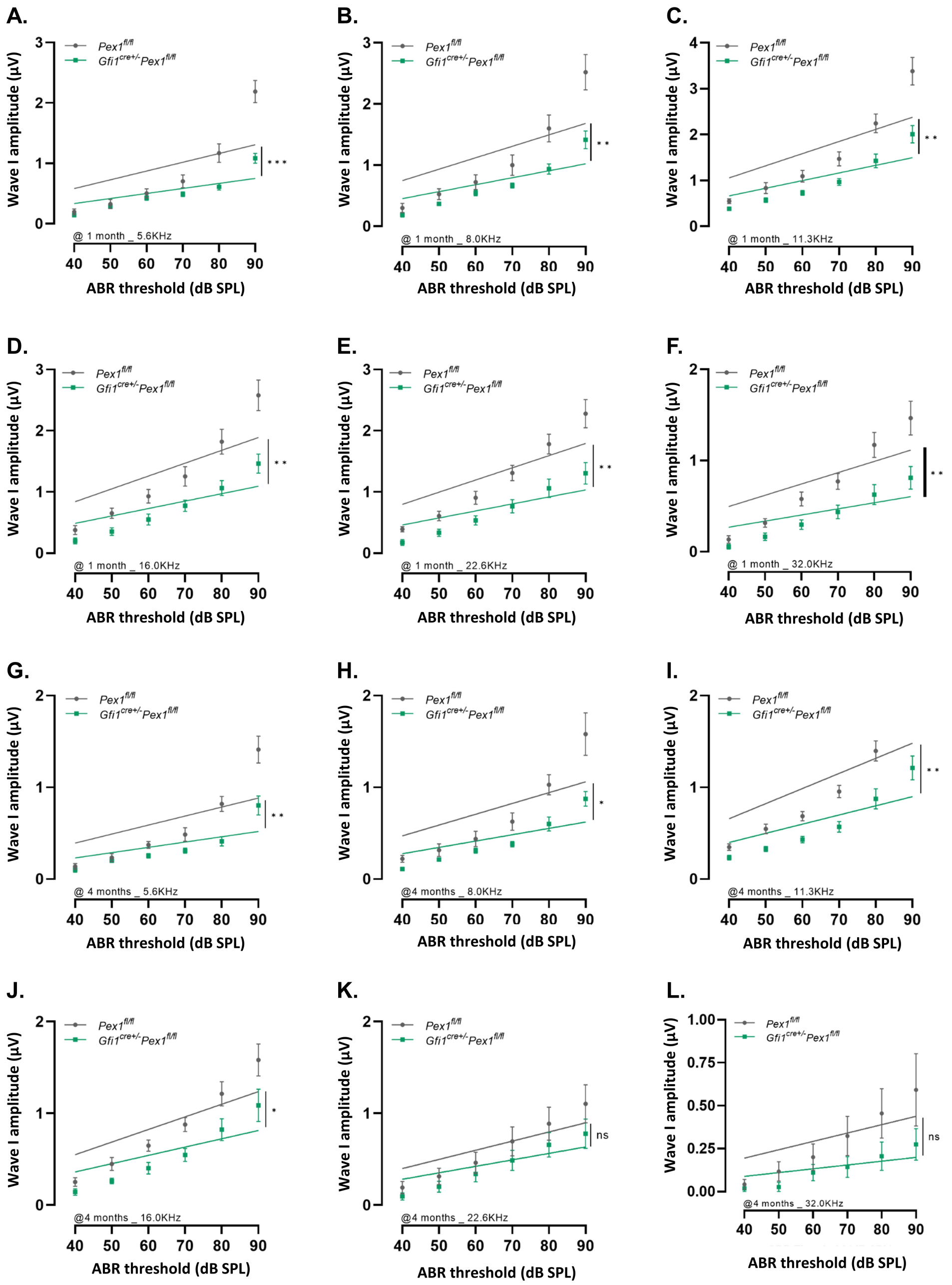
A.
Pex1fl/fl
Gfi1cre+/-Pex1fl/fl
Wave I amplitude (µV)
@ 1 month _ 5.6KHz
***
ABR threshold (dB SPL)
B.
Pex1fl/fl
Gfi1cre+/-Pex1fl/fl
Wave I amplitude (µV)
@ 1 month _ 8.0KHz
**
ABR threshold (dB SPL)
C.
Pex1fl/fl
Gfi1cre+/-Pex1fl/fl
Wave I amplitude (µV)
@ 1 month _ 11.3KHz
**
ABR threshold (dB SPL)
D.
Pex1fl/fl
Gfi1cre+/-Pex1fl/fl
Wave I amplitude (µV)
@ 1 month _ 16.0KHz
**
ABR threshold (dB SPL)
E.
Pex1fl/fl
Gfi1cre+/-Pex1fl/fl
Wave I amplitude (µV)
@ 1 month _ 22.6KHz
**
ABR threshold (dB SPL)
F.
Pex1fl/fl
Gfi1cre+/-Pex1fl/fl
Wave I amplitude (µV)
@ 1 month _ 32.0KHz
**
ABR threshold (dB SPL)
G.
Pex1fl/fl
Gfi1cre+/-Pex1fl/fl
Wave I amplitude (µV)
@ 4 months _ 5.6KHz
**
ABR threshold (dB SPL)
H.
Pex1fl/fl
Gfi1cre+/-Pex1fl/fl
Wave I amplitude (µV)
@4 months _ 8.0KHz
*
ABR threshold (dB SPL)
I.
Pex1fl/fl
Gfi1cre+/-Pex1fl/fl
Wave I amplitude (µV)
@4 months _ 11.3KHz
**
ABR threshold (dB SPL)
J.
Pex1fl/fl
Gfi1cre+/-Pex1fl/fl
Wave I amplitude (µV)
@4 months _ 16.0KHz
*
ABR threshold (dB SPL)
K.
Pex1fl/fl
Gfi1cre+/-Pex1fl/fl
Wave I amplitude (µV)
@4 months _ 22.6KHz
ns
ABR threshold (dB SPL)
L.
Pex1fl/fl
Gfi1cre+/-Pex1fl/fl
Wave I amplitude (µV)
@4 months _ 32.0KHz
ns
ABR threshold (dB SPL)

Figure S2: Decrease of the wave I amplitude observed over age during the recording of auditory brainstem responses (ABRs) *in Gfi1$^{cre/+}$Pex1$^{fl/fl}$* mice compared to control (*Pex1$^{fl/fl}$*).

(A-F) Wave I amplitude (µV) as a function of ABRs threshold (dB sounds pressure level (SPL)) of pure tones recording at 5.6 **(A),** 8.0 **(B)**, 11.3 **(C),** 16.0 **(D),** 22.6 **(E),** and 32.0 KHz **(F)** at 1 month (table S17 - Mean ± S.E.M. – 5.6KHz: *** $p = 0.0005$, 8.0KHz: ** $p = 0.0048$, 11.3KHz: ** $p = 0.0024$, 16.0KHz: ** $p = 0.0036$, 22.6KHz ** $p = 0.0060$, 32.0KHz: ** $p = 0.0087$ – unpaired t-test - *Pex1$^{fl/fl}$* (grey, $n = 6$), *Gfi1$^{cre/+}$Pex1$^{fl/fl}$* (green, $n = 12$)); **(G-L)** Wave I amplitude (µV) as a function of ABRs threshold (dB sounds pressure level (SPL)) of pure tones recording at 5.6 **(G),** 8.0 **(H)**, 11.3 **(I),** 16.0 **(J),** 22.6 **(K),** and 32.0 KHz **(L)** at 4 months (table S17 - Mean ± S.E.M. – 5.6KHz: ** $p = 0.0037$, 8.0KHz: * $p = 0.0106$, 11.3KHz: ** $p = 0.0026$, 16.0KHz: * $p = 0.0270$, 22.6KHz ns $p = 0.2770$, 32.0KHz: ns $p = 0.1669$ – unpaired t-test - *Pex1$^{fl/fl}$* (grey, $n = 8$), *Gfi1$^{cre/+}$Pex1$^{fl/fl}$* (green, $n = 9$)).The solid line represents the simple linear regression.

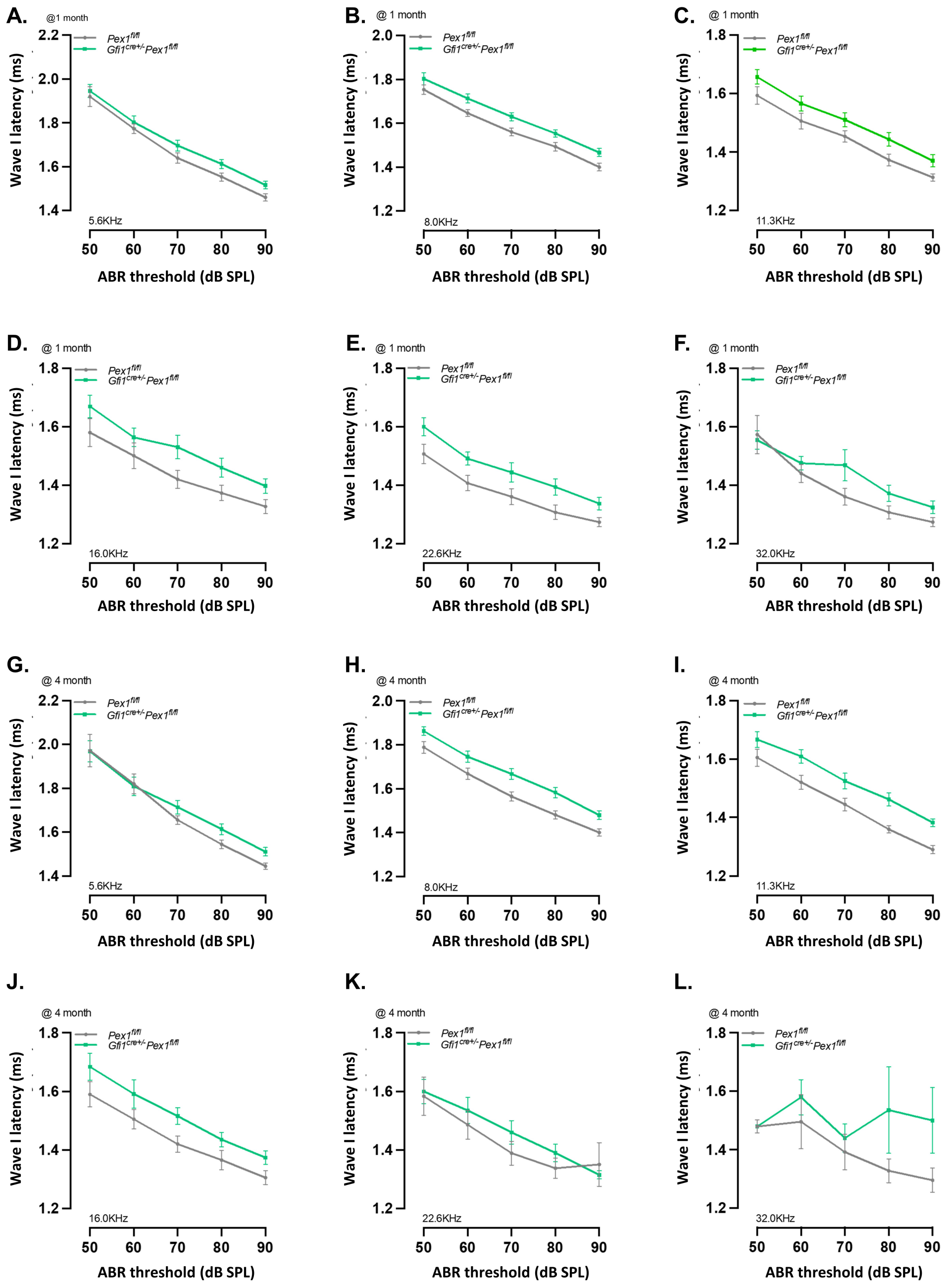
A.
@1 month
Pex1fl/fl
Gfi1cre+/-Pex1fl/fl
Wave I latency (ms)
2.2
2.0
1.8
1.6
1.4
5.6KHz
50
60
70
80
90
ABR threshold (dB SPL)
B.
@ 1 month
2.0
1.8
1.6
1.4
1.2
8.0KHz
C.
@ 1 month
1.8
1.6
1.4
1.2
11.3KHz
D.
@ 1 month
16.0KHz
E.
@ 1 month
22.6KHz
F.
@ 1 month
32.0KHz
G.
@ 4 month
5.6KHz
H.
@ 4 month
8.0KHz
I.
@ 4 month
11.3KHz
J.
@ 4 month
16.0KHz
K.
@ 4 month
22.6KHz
L.
@ 4 month
32.0KHz

<u>Figure S3:</u> Slight increase of the wave I latency measured on ABR recordings *in Gfi1$^{cre/+}$Pex1$^{fl/fl}$* mice compared to control (*Pex1$^{fl/fl}$*).
(A-F) Wave I latency (ms) as a function of ABRs threshold (dB sounds pressure level (SPL)) of pure tones recording at 5.6 **(A),** 8.0 **(B)**, 11.3 **(C),** 16.0 **(D),** 22.6 **(E),** and 32.0 KHz **(F)** at 1 month (Mean ± S.E.M. - *Pex1$^{fl/fl}$* (grey, *n* = 6), *Gfi1$^{cre/+}$Pex1$^{fl/fl}$* (green, *n* = 12); **(G-L)** Wave I latency (ms) as a function of ABRs threshold (dB sounds pressure level (SPL)) of pure tones recording at 5.6 **(G),** 8.0 **(H)**, 11.3 **(I),** 16.0 **(J),** 22.6 **(K),** and 32.0 KHz **(L)** at 4 months (Mean ± S.E.M. - *Pex1$^{fl/fl}$* (grey, *n* = 6), *Gfi1$^{cre/+}$Pex1$^{fl/fl}$* (green, *n* = 12)).

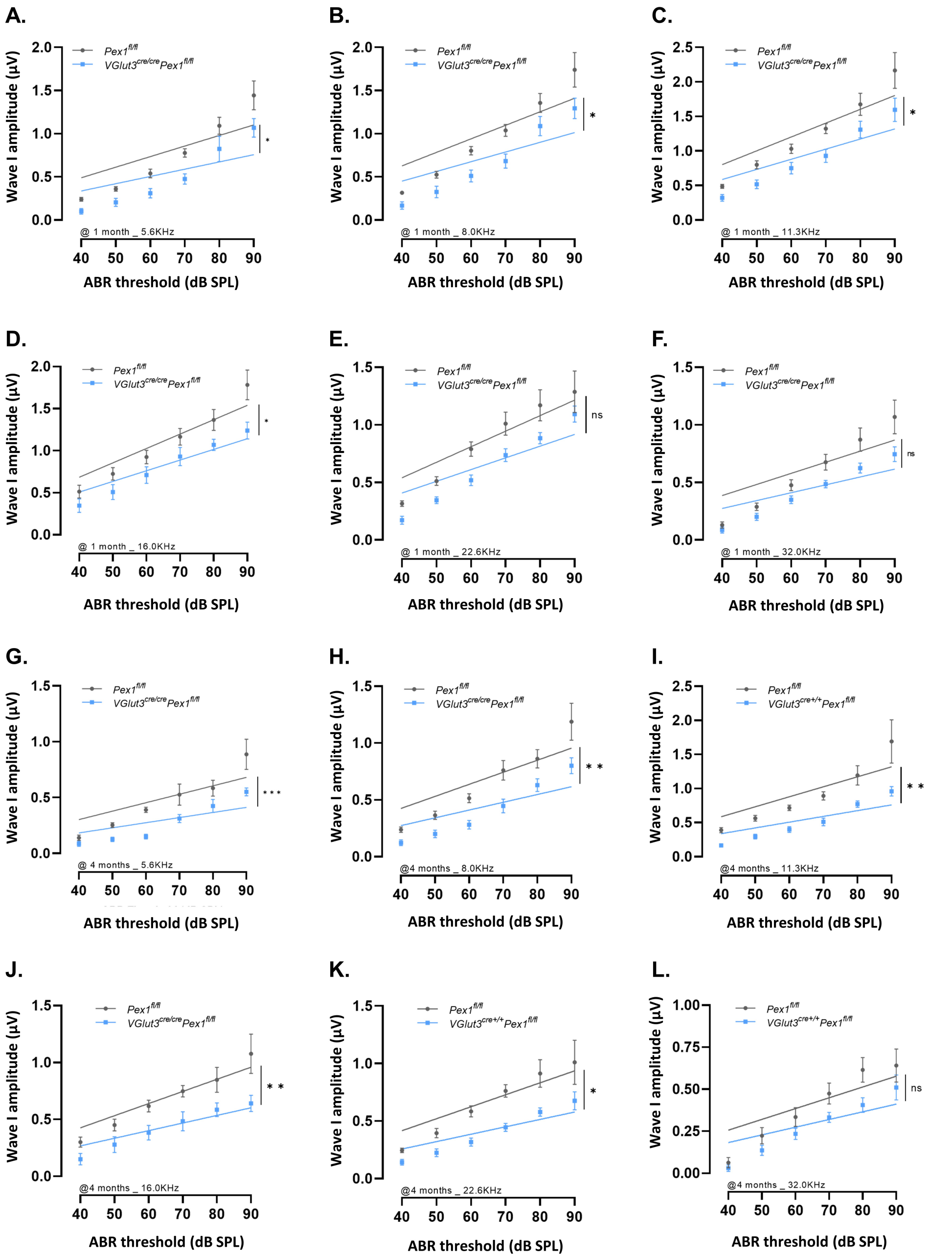
A.
Pex1fl/fl
VGlut3cre/crePex1fl/fl
Wave I amplitude (µV)
@ 1 month _ 5.6KHz
ABR threshold (dB SPL)
B.
Pex1fl/fl
VGlut3cre/crePex1fl/fl
Wave I amplitude (µV)
@ 1 month _ 8.0KHz
ABR threshold (dB SPL)
*
C.
Pex1fl/fl
VGlut3cre/crePex1fl/fl
Wave I amplitude (µV)
@ 1 month _ 11.3KHz
ABR threshold (dB SPL)
*
D.
Pex1fl/fl
VGlut3cre/crePex1fl/fl
Wave I amplitude (µV)
@ 1 month _ 16.0KHz
ABR threshold (dB SPL)
E.
Pex1fl/fl
VGlut3cre/crePex1fl/fl
Wave I amplitude (µV)
@ 1 month _ 22.6KHz
ABR threshold (dB SPL)
ns
F.
Pex1fl/fl
VGlut3cre/crePex1fl/fl
Wave I amplitude (µV)
@ 1 month _ 32.0KHz
ABR threshold (dB SPL)
ns
G.
Pex1fl/fl
VGlut3cre/crePex1fl/fl
Wave I amplitude (µV)
@ 4 months _ 5.6KHz
ABR threshold (dB SPL)
H.
Pex1fl/fl
VGlut3cre/crePex1fl/fl
Wave I amplitude (µV)
@4 months _ 8.0KHz
ABR threshold (dB SPL)
* *
I.
Pex1fl/fl
VGlut3cre+/+Pex1fl/fl
Wave I amplitude (µV)
@4 months _ 11.3KHz
ABR threshold (dB SPL)
* *
J.
Pex1fl/fl
VGlut3cre/crePex1fl/fl
Wave I amplitude (µV)
@4 months _ 16.0KHz
ABR threshold (dB SPL)
* *
K.
Pex1fl/fl
VGlut3cre+/+Pex1fl/fl
Wave I amplitude (µV)
@4 months _ 22.6KHz
ABR threshold (dB SPL)
*
L.
Pex1fl/fl
VGlut3cre+/+Pex1fl/fl
Wave I amplitude (µV)
@4 months _ 32.0KHz
ABR threshold (dB SPL)
ns

Figure S4: Decrease of the wave I amplitude observed over age during ABR recordings *in VGlut3$^{cre/cre}$Pex1$^{fl/fl}$* mice compared to control (*Pex1$^{fl/fl}$*).

(A-F) Wave I amplitude (µV) as a function of ABRs threshold (dB sounds pressure level (SPL)) of pure tones recording at 5.6 **(A),** 8.0 **(B)**, 11.3 **(C),** 16.0 **(D),** 22.6 **(E),** and 32.0 KHz **(F)** at 1 month (table S18 - Mean ± S.E.M. – 5.6KHz: * p = 0.0174, 8.0KHz: * p = 0.0175, 11.3KHz: * p = 0.0283, 16.0KHz: * p = 0.00265, 22.6KHz ns p = 0.0668, 32.0KHz: ns p = 0.0544 – unpaired t-test - *Pex1$^{fl/fl}$* (grey, n = 17), *VGlut3$^{cre/cre}$Pex1$^{fl/fl}$* (blue, n = 12)); **(G-L)** Wave I amplitude (µV) as a function of ABRs Threshold (dB sounds pressure level (SPL)) of pure tones recording at 5.6 **(G),** 8.0 **(H)**, 11.3 **(I),** 16.0 **(J),** 22.6 **(K),** and 32.0 KHz **(L)** at 4 months (table S18 - Mean ± S.E.M. – 5.6KHz: *** p = 0.0007, 8.0KHz: ** p = 0.0014, 11.3KHz: ** p = 0.0028, 16.0KHz: ** p = 0.0040, 22.6KHz * p = 0.0101, 32.0KHz: ns p = 0.0807 – unpaired t-test - *Pex1$^{fl/fl}$* (grey, n = 8), *VGlut3$^{cre/cre}$Pex1$^{fl/fl}$* (blue, n = 9)). The solid line represents the simple linear regression.

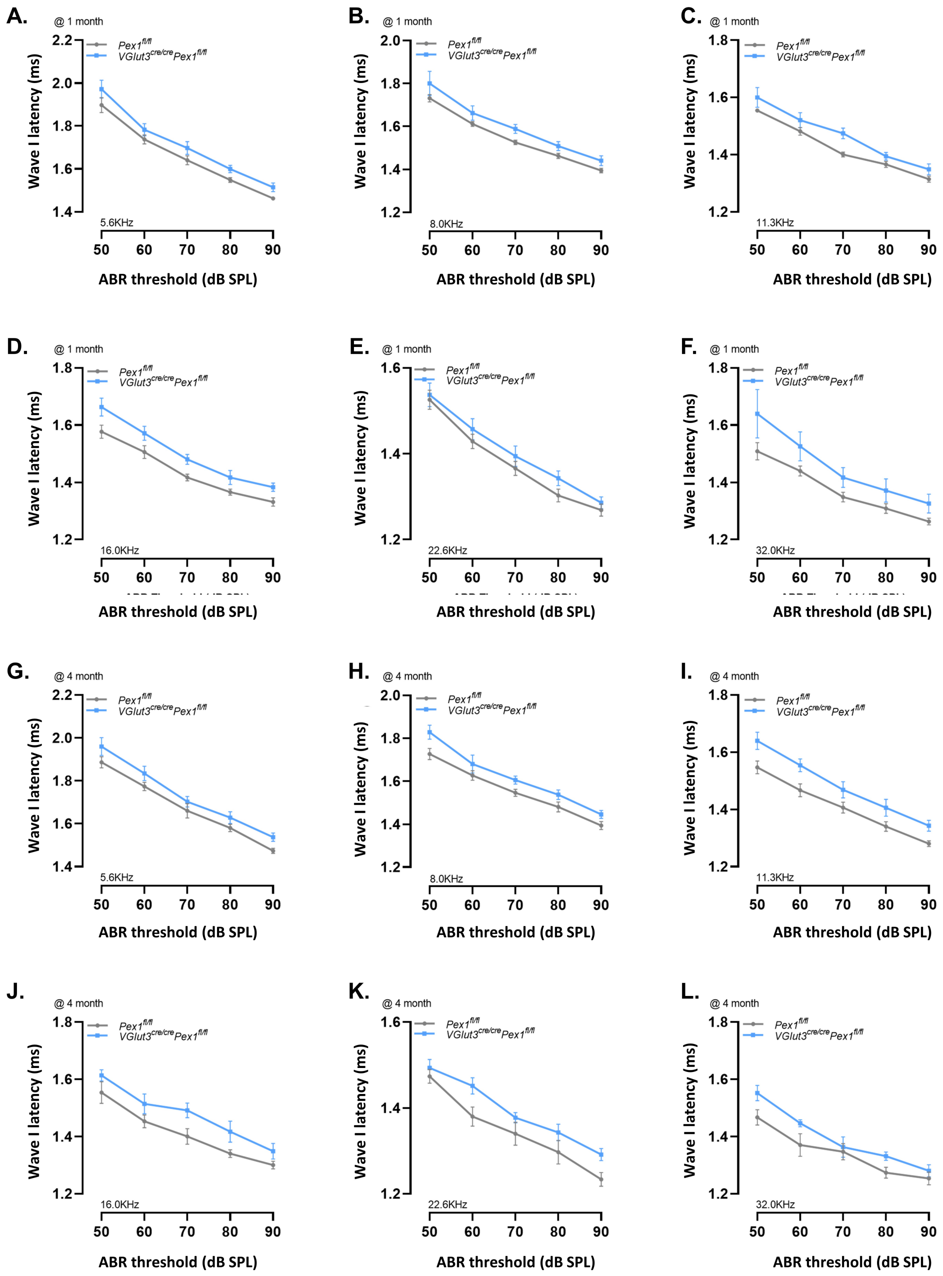
A.
B.
C.
D.
E.
F.
G.
H.
I.
J.
K.
L.
@ 1 month
@ 4 month
Pex1fl/fl
VGlut3cre/crePex1fl/fl
Wave I latency (ms)
ABR threshold (dB SPL)
5.6KHz
8.0KHz
11.3KHz
16.0KHz
22.6KHz
32.0KHz

Figure S5: **Slight elevation of the wave I latency measured on ABR recordings in *VGlut3*$^{cre/cre}$*Pex1*$^{fl/fl}$ mice compared to control (*Pex1*$^{fl/fl}$).** **(A-F)** Wave I latency (ms) as a function of ABRs threshold (dB sounds pressure level (SPL)) of pure tones recording at 5.6 **(A),** 8.0 **(B)**, 11.3 **(C),** 16.0 **(D),** 22.6 **(E),** and 32.0 KHz **(F)** at 1 month (*Pex1*$^{fl/fl}$ (grey) *n* = 17, *VGlut3*$^{cre/cre}$*Pex1*$^{fl/fl}$ (blue) *n* = 12; **(G-L)** Wave I latency (ms) as a function of ABRs threshold (dB sounds pressure level (SPL)) of pure tones recording at 5.6 **(G),** 8.0 **(H)**, 11.3 **(I),** 16.0 **(J),** 22.6 **(K),** and 32.0 KHz **(L)** at 4 months (*Pex1*$^{fl/fl}$ (grey) *n* = 9, *VGlut3*$^{cre/cre}$*Pex1*$^{fl/fl}$ (blue) *n* = 10). Mean ± S.E.M.

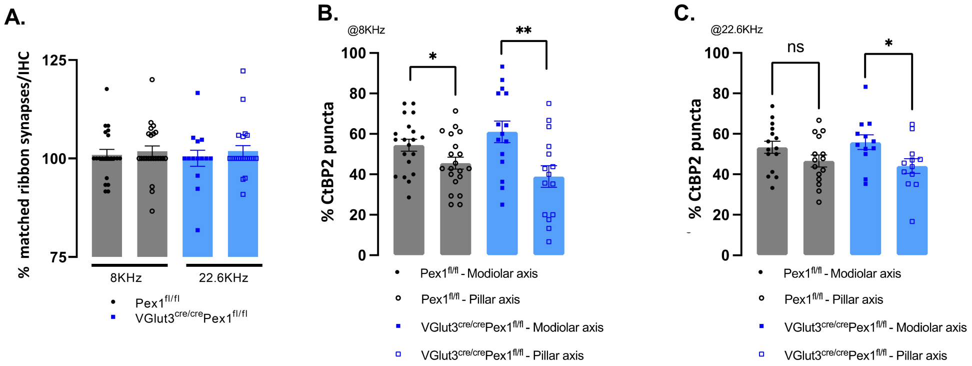

A.
% matched ribbon synapses/IHC
125
100
75
8KHz
22.6KHz
Pex1fl/fl
VGlut3cre/crePex1fl/fl
B.
@8KHz
% CtBP2 puncta
100
80
60
40
20
0
*
**
Pex1fl/fl - Modiolar axis
Pex1fl/fl - Pillar axis
VGlut3cre/crePex1fl/fl - Modiolar axis
VGlut3cre/crePex1fl/fl - Pillar axis
C.
@22.6KHz
% CtBP2 puncta
100
80
60
40
20
0
ns
*
Pex1fl/fl - Modiolar axis
Pex1fl/fl - Pillar axis
VGlut3cre/crePex1fl/fl - Modiolar axis
VGlut3cre/crePex1fl/fl - Pillar axis

Figure S6: Unchanged distribution of ribbon synapses along Modiolar/Pillar axis in *VGlut3$^{cre/cre}$Pex1$^{fl/fl}$* compared to control mice (*Pex1$^{fl/fl}$*)

(A) Percentage of matched ribbon synapses per IHC at 8 and 22.6 KHz (table S19 – at 8 KHz: ns $p > 0.999$; *Pex1$^{fl/fl}$* (mice: $n = 6$ – IHC: $n = 21$), *VGlut3$^{cre/cre}$Pex1$^{fl/fl}$ (mice: $n = 6$ – IHC: $n = 23$), at* 22.6 KHz *Pex1$^{fl/fl}$* (mice: $n = 4$ – IHC: $n = 14$), *VGlut3$^{cre/cre}$Pex1$^{fl/fl}$* (mice: $n = 6$ – IHC: $n = 22$) – 2way ANOVA Bonferroni's multicomparison test; **(B-C)** Percentage of CtBP2 puncta per IHC along the Modiolar/Pillar axis at 8 **(B)** and 22.6 KHz **(C)** (table S19; 8 KHz: *Pex1$^{fl/fl}$* (mice: $n = 5$ – IHC: $n = 20$) * $p = 0.0420$, *VGlut3$^{cre/cre}$Pex1$^{fl/fl}$* (mice: $n = 4$ – IHC: $n = 15$) ** $p = 0.0066$ – *22.6KHz: Pex1$^{fl/fl}$* (mice: $n = 5$ – IHC: $n = 20$) *ns $p = 0.1272$, VGlut3$^{cre/cre}$Pex1$^{fl/fl}$* (mice: $n = 4$ – IHC: $n = 15$), * $p = 0.0320$. unpaired t-test with Welch's correction). Mean ± S.E.M.

Table S1

Figure 2A-D

			ABRs (db SPL)					
			Pex1 $^{fl/fl}$			*Gfi1* $^{cre/+}$ *Pex1* $^{fl/fl}$		
			n	Mean	S.E.M.	n	Mean	S.E.M.
1 month	**Click**		**6**	44.17	2.71	**12**	44.58	1.30
		statistical test	*Mann-Whitney test, ns p= 0.9334*					
	**5.6 kHz**		**6**	38.33	2.47	**12**	38.75	1.39
		statistical test	2way ANOVA Bonferroni's multicomparison test, ns P>0.999					
	**8 kHz**		**6**	32.50	1.71	**12**	35.83	1.72
		statistical test	2way ANOVA Bonferroni's multicomparison test, ns P>0.999					
	**11.3 kHz**		**6**	25.83	0.83	**12**	26.67	1.78
		statistical test	2way ANOVA Bonferroni's multicomparison test, ns P>0.999					
	**16 kHz**		**6**	26.67	2.11	**12**	37.08	3.51
		statistical test	2way ANOVA Bonferroni's multicomparison test, ns P=0.213					
	**22.6 kHz**		**6**	30.00	1.83	**12**	37.92	3.34
		statistical test	2way ANOVA Bonferroni's multicomparison test, ns P=0.631					
	**32 kHz**		**6**	40.00	2.24	**12**	53.75	5.61
		statistical test	2way ANOVA Bonferroni's multicomparison test, *** P=0.033**					
4 months	**Click**		**8**	45.63	1.99	**9**	46.67	1.86
		statistical test	*Mann-Whitney test, ns p= 0.9638*					
	**5.6 kHz**		**8**	38.13	2.30	**9**	40.00	1.44
		statistical test	2way ANOVA Bonferroni's multicomparison test, ns P>0.999					
	**8 kHz**		**8**	35.63	4.38	**9**	36.67	1.44
		statistical test	2way ANOVA Bonferroni's multicomparison test, ns P>0.999					
	**11.3 kHz**		**8**	27.50	1.34	**9**	32.33	1.47
		statistical test	2way ANOVA Bonferroni's multicomparison test, ns P>0.999					
	**16 kHz**		**8**	31.25	3.10	**9**	35.00	2.36
		statistical test	2way ANOVA Bonferroni's multicomparison test, ns P>0.999					
	**22.6 kHz**		**8**	42.50	7.38	**9**	43.33	3.73
		statistical test	2way ANOVA Bonferroni's multicomparison test, ns P>0.999					
	**32 kHz**		**8**	61.88	8.50	**9**	72.22	6.57
		statistical test	2way ANOVA Bonferroni's multicomparison test, ns P=0.551					

Table S2

Figure 2E-F

			DPOAEs (dB SPL)					
			Pex1 $^{fl/fl}$			***Gfi1*** $^{cre/+}$ ***Pex1*** $^{fl/fl}$		
			**n**	**Mean**	**S.E.M.**	**n**	**Mean**	**S.E.M.**
1 month	**5.6 kHz**		**6**	50.83	5.07	**12**	53.33	4.74
		statistical test	2way ANOVA Bonferroni's multicomparison test, ns P>0.999					
	**8 kHz**		**6**	36.67	3.80	**12**	39.58	3.17
		statistical test	2way ANOVA Bonferroni's multicomparison test, ns P>0.999					
	**11.3 kHz**		**6**	30.83	2.39	**12**	31.25	2.76
		statistical test	2way ANOVA Bonferroni's multicomparison test, ns P>0.999					
	**16 kHz**		**6**	27.50	2.81	**12**	30.42	3.67
		statistical test	2way ANOVA Bonferroni's multicomparison test, ns P>0.999					
	**22.6 kHz**		**6**	38.33	3.58	**12**	42.08	3.87
		statistical test	2way ANOVA Bonferroni's multicomparison test, ns P>0.999					
	**32 kHz**		**6**	43.33	3.58	**12**	54.58	4.90
		statistical test	2way ANOVA Bonferroni's multicomparison test, ns P=0.557					
	**45.2 kHz**		**6**	50.83	3.75	**12**	63.75	4.85
		statistical test	2way ANOVA Bonferroni's multicomparison test, ns P=0.312					
4 months	**5.6 kHz**		**8**	48.75	4.20	**9**	49.44	3.77
		statistical test	2way ANOVA Bonferroni's multicomparison test, ns P>0.999					
	**8 kHz**		**8**	36.25	3.75	**9**	39.44	2.82
		statistical test	2way ANOVA Bonferroni's multicomparison test, ns P>0.999					
	**11.3 kHz**		**8**	30.63	3.05	**9**	30.56	3.48
		statistical test	2way ANOVA Bonferroni's multicomparison test, ns P>0.999					
	**16 kHz**		**8**	29.38	2.58	**9**	30.00	4.25
		statistical test	2way ANOVA Bonferroni's multicomparison test, ns P>0.999					
	**22.6 kHz**		**8**	43.75	6.03	**9**	47.79	5.15
		statistical test	2way ANOVA Bonferroni's multicomparison test, ns P>0.999					
	**32 kHz**		**8**	58.13	6.94	**9**	66.11	5.32
		statistical test	2way ANOVA Bonferroni's multicomparison test, ns P>0.999					
	**45.2 kHz**		**8**	65.63	5.38	**9**	78.89	5.12
		statistical test	2way ANOVA Bonferroni's multicomparison test, ns P=0.300					

Table S3

Figure 2I-J

			Wave I amplitude (µV)					
			Pex1 $^{fl/fl}$			*Gfi1* $^{cre/+}$ *Pex1* $^{fl/fl}$		
			n	Mean	S.E.M.	n	Mean	S.E.M.
1 month	**5.6 kHz**		**6**	1.35	0.14	**12**	0.73	0.09
		statistical test	unpaired t-test with Welch's correction, **** P=0.0054**					
	**8 kHz**		**6**	1.71	0.22	**12**	1.01	0.09
		statistical test	unpaired t-test with Welch's correction, *** P=0.0235**					
	**11.3 kHz**		**6**	2.37	0.22	**12**	1.47	1.14
		statistical test	unpaired t-test with Welch's correction, **** P=0.0064**					
	**16 kHz**		**6**	1.89	0.20	**12**	1.10	0.12
		statistical test	unpaired t-test with Welch's correction, **** P=0.0084**					
	**22.6 kHz**		**6**	1.79	0.17	**12**	1.04	0.14
		statistical test	unpaired t-test with Welch's correction, **** P=0.0049**					
	**32 kHz**		**6**	1.14	0.13	**12**	0.62	0.10
		statistical test	unpaired t-test with Welch's correction, *** P=0.0109**					
4 months	**5.6 kHz**		**8**	0.91	0.09	**9**	0.51	0.06
		statistical test	unpaired t-test with Welch's correction, **** P=0.0034**					
	**8 kHz**		**8**	1.08	0.14	**9**	0.62	0.06
		statistical test	unpaired t-test with Welch's correction, *** P=0.0144**					
	**11.3 kHz**		**8**	1.47	0.14	**9**	0.89	0.10
		statistical test	unpaired t-test with Welch's correction, **** P=0.0042**					
	**16 kHz**		**8**	1.23	0.12	**9**	0.81	0.12
		statistical test	unpaired t-test with Welch's correction, *** P=0.0293**					
	**22.6 kHz**		**8**	0.90	0.18	**8**	0.72	0.12
		statistical test	unpaired t-test with Welch's correction, ns P=0.4393					
	**32 kHz**		**8**	1.06	0.10	**9**	0.96	0.07
		statistical test	unpaired t-test with Welch's correction, ns P=0.4436					

Table S4

Figure 2K-L

			Wave I latency (ms)					
			Pex1 fl/fl			*Gfi1 cre/+ Pex1 fl/fl*		
			n	Mean	S.E.M.	n	Mean	S.E.M.
1 month	**5.6 kHz**		**6**	1.54	0.02	**12**	1.61	0.02
		statistical test	unpaired t-test with Welch's correction, *** P=0.0341**					
	**8 kHz**		**6**	1.48	0.02	**12**	1.55	0.02
		statistical test	unpaired t-test with Welch's correction, *** P=0.0196**					
	**11.3 kHz**		**6**	1.38	0.02	**12**	1.44	0.02
		statistical test	unpaired t-test with Welch's correction, *** P=0.0451**					
	**16 kHz**		**6**	1.37	0.03	**12**	1.46	0.03
		statistical test	unpaired t-test with Welch's correction, ns P=0.0502					
	**22.6 kHz**		**6**	1.31	0.02	**12**	1.39	0.03
		statistical test	unpaired t-test with Welch's correction, *** P=0.0417**					
	**32 kHz**		**6**	1.31	0.02	**10**	1.39	0.03
		statistical test	unpaired t-test with Welch's correction, ns P=0.0807					
4 months	**5.6 kHz**		**8**	1.55	0.02	**9**	1.61	0.02
		statistical test	unpaired t-test with Welch's correction, *** P=0.0261**					
	**8 kHz**		**8**	1.48	0.01	**9**	1.58	0.21
		statistical test	unpaired t-test with Welch's correction, **** P=0.0034**					
	**11.3 kHz**		**8**	1.37	0.02	**9**	1.46	0.02
		statistical test	unpaired t-test with Welch's correction, **** P=0.0024**					
	**16 kHz**		**8**	1.36	0.03	**9**	1.44	0.03
		statistical test	unpaired t-test with Welch's correction, ns P=0.0547					
	**22.6 kHz**		**8**	1.40	0.07	**8**	1.39	0.03
		statistical test	unpaired t-test with Welch's correction, ns P=0.8970					
	**32 kHz**		**5**	1.34	0.05	**6**	1.57	0.12
		statistical test	unpaired t-test with Welch's correction, ns P=0.1122					

Table S5

Figure 3A-D

			ABRs (db SPL)					
			Pex1 $^{fl/fl}$			*VGlut3* $^{cre/cre}$ *Pex1* $^{fl/fl}$		
			**n**	**Mean**	**S.E.M.**	**n**	**Mean**	**S.E.M.**
1 month	**Click**		**17**	45.29	1.09	**12**	47.92	1.56
		statistical test	*Mann-Whitney test, ns p= 0.2243*					
	**5.6 kHz**		**17**	30.59	1.28	**12**	43.33	3.45
		statistical test	2way ANOVA Bonferroni's multicomparison test, ****** P=0.00007**					
	**8 kHz**		**17**	28.24	1.13	**12**	37.08	3.40
		statistical test	2way ANOVA Bonferroni's multicomparison test, *** P=0.01236**					
	**11.3 kHz**		**17**	23.82	0.68	**12**	28.75	2.23
		statistical test	2way ANOVA Bonferroni's multicomparison test, ns P=0.4983					
	**16 kHz**		**17**	23.24	2.39	**12**	29.58	2.78
		statistical test	2way ANOVA Bonferroni's multicomparison test, ns P=0.1559					
	**22.6 kHz**		**17**	29.71	0.91	**12**	35.42	2.17
		statistical test	2way ANOVA Bonferroni's multicomparison test, ns P=0.2691					
	**32 kHz**		**17**	38.53	1.41	**11**	42.27	1.95
		statistical test	2way ANOVA Bonferroni's multicomparison test, ns P>0.999					
4 months	**Click**		**10**	43.00	1.53	**9**	46.11	1.39
		statistical test	*Mann-Whitney test, ns p= 0.2962*					
	**5.6 kHz**		**10**	35.20	1.86	**9**	41.11	3.71
		statistical test	2way ANOVA Bonferroni's multicomparison test, ns P=0.1421					
	**8 kHz**		**10**	29.00	1.80	**9**	39.44	4.12
		statistical test	2way ANOVA Bonferroni's multicomparison test, *** P=0.0382**					
	**11.3 kHz**		**10**	23.50	1.50	**9**	33.33	2.76
		statistical test	2way ANOVA Bonferroni's multicomparison test, ns P=0.0604					
	**16 kHz**		**10**	26.50	2.69	**9**	37.78	3.45
		statistical test	2way ANOVA Bonferroni's multicomparison test, * **P=0.0199**					
	**22.6 kHz**		**10**	31.00	1.45	**9**	38.89	2.32
		statistical test	2way ANOVA Bonferroni's multicomparison test, ns P=0.2271					
	**32 kHz**		**10**	41.00	2.77	**9**	46.67	2.64
		statistical test	2way ANOVA Bonferroni's multicomparison test, ns P=0.8029					

Table S6

Figure 3E-F

			DPOAEs (dB SPL)					
			Pex1 fl/fl			*VGlut3 cre/cre Pex1 fl/fl*		
			**n**	**Mean**	**S.E.M.**	**n**	**Mean**	**S.E.M.**
1 month	**5.6 kHz**		**17**	54.12	3.04	**12**	60.83	2.81
		statistical test	2way ANOVA Bonferroni's multicomparison test, ns P=0.5657					
	**8 kHz**		**17**	40.88	1.62	**12**	45.83	4.52
		statistical test	2way ANOVA Bonferroni's multicomparison test, ns P>0.999					
	**11.3 kHz**		**17**	31.47	1.41	**12**	34.58	3.67
		statistical test	2way ANOVA Bonferroni's multicomparison test, ns P>0.999					
	**16 kHz**		**17**	30.59	1.28	**12**	32.50	2.58
		statistical test	2way ANOVA Bonferroni's multicomparison test, ns P>0.999					
	**22.6 kHz**		**17**	40.00	2.01	**12**	42.92	2.66
		statistical test	2way ANOVA Bonferroni's multicomparison test, ns P>0.999					
	**32 kHz**		**17**	46.76	2.14	**12**	52.50	3.62
		statistical test	2way ANOVA Bonferroni's multicomparison test, ns P=0.9486					
	**45.2 kHz**		**17**	52.06	2.68	**12**	57.50	3.82
		statistical test	2way ANOVA Bonferroni's multicomparison test, ns P>0.999					
4 months	**5.6 kHz**		**10**	51.00	2.56	**9**	48.89	4.06
		statistical test	2way ANOVA Bonferroni's multicomparison test, ns P>0.999					
	**8 kHz**		**10**	37.00	2.38	**9**	34.44	3.86
		statistical test	2way ANOVA Bonferroni's multicomparison test, ns P>0.999					
	**11.3 kHz**		**10**	28.50	2.24	**9**	30.56	2.56
		statistical test	2way ANOVA Bonferroni's multicomparison test, ns P>0.999					
	**16 kHz**		**10**	28.00	2.00	**9**	30.56	2.82
		statistical test	2way ANOVA Bonferroni's multicomparison test, ns P>0.999					
	**22.6 kHz**		**10**	41.50	2.24	**9**	41.11	3.51
		statistical test	2way ANOVA Bonferroni's multicomparison test, ns P>0.999					
	**32 kHz**		**10**	52.00	3.09	**9**	45.00	4.00
		statistical test	2way ANOVA Bonferroni's multicomparison test, ns P=0.8351					
	**45.2 kHz**		**10**	55.00	3.87	**9**	51.67	4.25
		statistical test	2way ANOVA Bonferroni's multicomparison test, ns P>0.999					

Table S7

Figure 4C-D

			Wave I amplitude (µV)					
			Pex1 fl/fl			*VGlut3 cre/cre Pex1 fl/fl*		
			**n**	**Mean**	**S.E.M.**	**n**	**Mean**	**S.E.M.**
1 month	**Click**		**7**	3.39	0.31	**7**	2.55	0.32
		statistical test	unpaired t-test with Welch's correction, ns P=0.0842					
	**5.6 kHz**		**7**	1.29	0.11	**7**	0.79	0.05
		statistical test	unpaired t-test with Welch's correction, **<u>** P=0.0028</u>**					
	**8 kHz**		**7**	1.78	0.10	**7**	1.18	0.09
		statistical test	unpaired t-test with Welch's correction, **<u>*** P=0.0009</u>**					
	**11.3 kHz**		**7**	2.28	0.15	**7**	1.37	0.13
		statistical test	unpaired t-test with Welch's correction, **<u>*** P=0.0007</u>**					
	**16 kHz**		**7**	1.62	0.18	**7**	1.00	0.04
		statistical test	unpaired t-test with Welch's correction, **<u>* P=0.0141</u>**					
	**22.6 kHz**		**7**	1.62	0.13	**7**	0.97	0.04
		statistical test	unpaired t-test with Welch's correction, **<u>** P=0.0022</u>**					
	**32 kHz**		**7**	1.16	0.15	**7**	0.69	0.05
		statistical test	unpaired t-test with Welch's correction, **<u>* P=0.0213</u>**					
4 months	**Click**		**6**	2.65	0.17	**7**	1.05	0.03
		statistical test	unpaired t-test with Welch's correction, **<u>*** P=0.0002</u>**					
	**5.6 kHz**		**6**	0.68	0.06	**7**	0.39	0.02
		statistical test	unpaired t-test with Welch's correction, **<u>* P=0.0048</u>**					
	**8 kHz**		**6**	1.03	0.06	**7**	0.63	0.04
		statistical test	unpaired t-test with Welch's correction, **<u>*** P=0.0002</u>**					
	**11.3 kHz**		**6**	1.53	0.12	**7**	0.75	0.03
		statistical test	unpaired t-test with Welch's correction, **<u>*** P=0.0009</u>**					
	**16 kHz**		**6**	1.02	0.11	**7**	0.55	0.04
		statistical test	unpaired t-test with Welch's correction, **<u>** P=0.0069</u>**					
	**22.6 kHz**		**6**	1.09	0.10	**7**	0.60	0.04
		statistical test	unpaired t-test with Welch's correction, **<u>** P=0.0036</u>**					
	**32 kHz**		**6**	0.68	0.07	**7**	0.45	0.04
		statistical test	unpaired t-test with Welch's correction, **<u>* P=0.0368</u>**					

Table S8

Figure 4E-F

			**Wave I latency (ms)**					
			Pex1 $^{fl/fl}$			***VGlut3*** $^{cre/cre}$ ***Pex1*** $^{fl/fl}$		
			**n**	**Mean**	**S.E.M.**	**n**	**Mean**	**S.E.M.**
1 month	**Click**		**7**	1.16	0.01	**7**	1.17	0.01
		statistical test	Mann-Whitney test, ns P=0.9825					
	**5.6 kHz**		**7**	1.55	0.01	**7**	1.60	0.02
		statistical test	*Mann-Whitney test,* **** p=0.0210***					
	**8 kHz**		**7**	1.46	0.01	**7**	1.51	0.02
		statistical test	*Mann-Whitney test,* **** p=0.0390***					
	**11.3 kHz**		**7**	1.37	0.01	**7**	1.41	0.02
		statistical test	*Mann-Whitney test,* **** p=0.0175***					
	**16 kHz**		**7**	1.37	0.01	**7**	1.43	0.02
		statistical test	*Mann-Whitney test,* **** p=0.0146***					
	**22.6 kHz**		**7**	1.31	0.01	**7**	1.34	0.02
		statistical test	*Mann-Whitney test, ns p= 0.2978*					
	**32 kHz**		**7**	1.31	0.01	**7**	1.34	0.02
		statistical test	*Mann-Whitney test, ns p= 0.2477*					
4 months	**Click**		**6**	1.12	0.02	**7**	1.18	0.04
		statistical test	Mann-Whitney test, ns P=0.3415					
	**5.6 kHz**		**6**	1.57	0.02	**7**	1.62	0.02
		statistical test	*Mann-Whitney test, ns p= 0.0822*					
	**8 kHz**		**6**	1.47	0.02	**7**	1.53	0.02
		statistical test	*Mann-Whitney test, ns p= 0.0629*					
	**11.3 kHz**		**6**	1.34	0.02	**7**	1.41	0.02
		statistical test	*Mann-Whitney test, ns p= 0.0565*					
	**16 kHz**		**6**	1.35	0.02	**7**	1.42	0.03
		statistical test	*Mann-Whitney test, ns p= 0.0542*					
	**22.6 kHz**		**6**	1.29	0.02	**7**	1.34	0.01
		statistical test	*Mann-Whitney test, ns p= 0.1241*					
	**32 kHz**		**6**	1.29	0.02	**7**	1.32	0.02
		statistical test	*Mann-Whitney test, ns p= 0.3910*					

Table S9

Figure 4K-L

			Wave IV amplitude (µV)					
			Pex1 $^{fl/fl}$			*VGlut3* $^{cre/cre}$ *Pex1* $^{fl/fl}$		
			**n**	**Mean**	**S.E.M.**	**n**	**Mean**	**S.E.M.**
1 month	**Click**		**7**	2.31	0.19	**7**	1.80	0.21
		statistical test	unpaired t-test with Welch's correction, ns P=0.0943					
4 months	**Click**		**6**	1.90	0.22	**7**	1.18	0.14
		statistical test	unpaired t-test with Welch's correction, *** P =0.0217**					

Table S10

Figure 4M-N

			Wave IV latency (ms)					
			Pex1 $^{fl/fl}$			*VGlut3* $^{cre/cre}$ *Pex1* $^{fl/fl}$		
				**Mean**	**S.E.M.**		**Mean**	**S.E.M.**
1 month	**Click**		**7**	3.76	0.04	**7**	3.74	0.03
		statistical test	unpaired t-test with Welch's correction, ns P=0.8269					
4 months	**Click**		**6**	3.47	0.05	**7**	3.55	0.07
		statistical test	unpaired t-test with Welch's correction, ns P =0.1346					

Table S11

Figure 4O-P

			ratio wave IV/I amplitude					
			Pex1 $^{fl/fl}$			*VGlut3* $^{cre/cre}$ *Pex1* $^{fl/fl}$		
				**Mean**	**S.E.M.**		**Mean**	**S.E.M.**
1 month	**Click**		**7**	0.73	0.10	**7**	0.73	0.09
		statistical test	unpaired t-test with Welch's correction, ns P=0.9839					
4 months	**Click**		**6**	0.74	0.10	**7**	1.11	0.10
		statistical test	unpaired t-test with Welch's correction, *** P =0.0288**					

Table S12

Figure 5C-F

		CtBP2 puncta/IHC							
		Pex1 $^{fl/fl}$				*VGlut3* $^{cre/cre}$ *Pex1* $^{fl/fl}$			
		**n (mice)**	**n (IHC)**	**Mean**	**S.E.M.**	**n (mice)**	**n (IHC)**	**Mean**	**S.E.M.**
8 kHz		**6**	**23**	14.43	0.51	**6**	**23**	14.35	0.39
	statistical test	Mann-Whitney test, ns P=0.9513							
22.6 kHz		**5**	**15**	19.81	0.67	**5**	**19**	18.05	0.55
	statistical test	Mann-Whitney test, ns P=0.0796							

		GluR2 puncta/IHC							
		Pex1 $^{fl/fl}$				*VGlut3* $^{cre/cre}$ *Pex1* $^{fl/fl}$			
		**n (mice)**	**n (IHC)**	**Mean**	**S.E.M.**	**n (mice)**	**n (IHC)**	**Mean**	**S.E.M.**
8 kHz		**6**	**23**	14.70	0.54	**6**	**23**	14.61	0.45
	statistical test	Mann-Whitney test, ns P=0.9866							
22.6 kHz		**4**	**14**	20.07	0.63	**5**	**19**	18.26	0.61
	statistical test	Mann-Whitney test, ns P=0.0524							

Table S13

Figure 5G-H

		Ribbon synapses volume (µm^3)							
		Pex1 $^{fl/fl}$				*VGlut3* $^{cre/cre}$ *Pex1* $^{fl/fl}$			
		**n (mice)**	**n (ribbon)**	**Mean**	**S.E.M.**	**n (mice)**	**n (ribbon)**	**Mean**	**S.E.M.**
8 kHz		**6**	**282**	0.49	0.03	**6**	**311**	0.32	0.02
	statistical test	Mann-Whitney test, ****** P<0.0001**							
22.6 kHz		**5**	**286**	0.52	0.02	**5**	**328**	0.31	0.02
	statistical test	Mann-Whitney test, ****** P<0.0001**							

Figure 5I-J

		% of CtBP2 punta							
		Pex1 $^{fl/fl}$				*VGlut3* $^{cre/cre}$ *Pex1* $^{fl/fl}$			
		**n (mice)**	**n (IHC)**	**n (ribbon)**	**Mean**	**n (mice)**	**n (IHC)**	**n (ribbon)**	**Mean**
8 KHz	*0-0.25*	**5**	25	**282**	37.23	**6**	22	**311**	53.05
	0.25-0.5				27.30				28.30
	0.5-1				23.05				14.79
	1-1.5				9.22				2.57
	1.5-2				1.77				0.96
	>2				1.42				0.32
22.6 kHz	*0-0.25*	**5**	15	**286**	26.57	**6**	19	**328**	60.98
	0.25-0.5				32.52				18.29
	0.5-1				32.17				13.41
	1-1.5				5.94				2.49
	1.5-2				1.75				1.83
	>2				1.05				0

Table S14

Figure 6

			Pex1 $^{fl/fl}$			*VGlut3* $^{cre/cre}$ *Pex1* $^{fl/fl}$				
			n	Mean	± S.E.M.	n	Mean	± S.E.M.		Significant
Ca^{2+}	Ramp calcique	Vhalf (mV)	17	-21.3657	0.66931	21	-21.27	0.68504	unpaired t-test : 0,93	ns
		$I_{Ca^{2+}}$ max (pA)	14	-138.85714	6.28521	17	-118.41176	5,88684	unpaired t-test : 0,025	*
	Resting cell size (pF)		17	10,82647□	0.35056	21	10.48571	0.3155	unpaired t-test : 0,47	ns
	Exocytosis	RRP (kinetics) curve	10			15			two-way anova / factor phenotype : 7,6E-8	***
		100ms stimulation (sustained, fF)	14	26,92857□	3.25722	17	15,92941	2.10833	Unpaired t-test : 0,00656	**
		Efficiency (fF/pA	14	-0.19405	0.01992	17	-0,13239	0.01426	Unpaired t-test :0,015	*
K^{+}	IV curve		14			15			two-way anova / factor phenotype : 0,65	ns

Table S15

Figure 7B

		Relative expression PMP70 protein					
		Pex1fl/fl			*VGlut3$^{cre/cre}$ Pex1$^{fl/fl}$*		
		**n (mice)**	**Mean**	**S.E.M.**	**n (mice)**	**Mean**	**S.E.M.**
8 kHz		**4**	100.00	1.66	**8**	66.95	4.14
	statistical test	unpaired t-test with Welch's correction, **** P<0.0001					

Table S16

Figure S1C

	Relative expression Pex1 protein					
	Pex1 $^{fl/fl}$			*Gfi1* $^{cre/+}$ *Pex1* $^{fl/fl}$		
	**n (mice)**	**Mean**	**S.E.M.**	**n (mice)**	**Mean**	**S.E.M.**
	**3**	100.00	26.84	**5**	86.22	17.11
statistical test	unpaired t-test with Welch's correction, ns P=0.6893					

Figure S1D

	Relative expression Pex1 protein					
	Pex1 $^{fl/fl}$			***VGlut3*** $^{cre/cre}$ ***Pex1*** $^{fl/fl}$		
	**n (mice)**	**Mean**	**S.E.M.**	**n (mice)**	**Mean**	**S.E.M.**
	**4**	100.00	6.81	**8**	13.88	3.54
statistical test	unpaired t-test with Welch's correction, *** P=0.0001					

Table S17

Figure S2A-L

			Slope wave I amplitude					
			Pex1 $^{fl/fl}$			*Gfi1* $^{cre/+}$ *Pex1* $^{fl/fl}$		
			**n**	**Mean**	**S.E.M.**	**n**	**Mean**	**S.E.M.**
1 month	**5.6 kHz**		**6**	0.145	0.002	**12**	0.008	0.001
		statistical	unpaired T-test, *** P=0.0005					
	**8 kHz**		**6**	0.019	0.003	**12**	0.011	0.001
		statistical	unpaired T-test, ** P=0.0048					
	**11.3 kHz**		**6**	0.026	0.002	**12**	0.017	0.001
		statistical	unpaired T-test, ** P=0.0024					
	**16 kHz**		**6**	0.021	0.002	**12**	0.012	0.001
		statistical	unpaired T-test, ** P=0.0036					
	**22.6 kHz**		**6**	0.020	0.002	**12**	0.011	0.002
		statistical	unpaired T-test, ** P=0.0060					
	**32 kHz**		**6**	0.012	0.001	**12**	0.007	0.001
		statistical	unpaired T-test, ** P=0.0087					
4 months	**5.6 kHz**		**8**	0.010	0.001	**9**	0.006	0.001
		statistical	unpaired T-test, ** P=0.0037					
	**8 kHz**		**8**	0.012	0.002	**9**	0.007	0.001
		statistical	unpaired T-test, * P=0.0106					
	**11.3 kHz**		**8**	0.016	0.002	**9**	0.010	0.001
		statistical	unpaired T-test, ** P=0.0026					
	**16 kHz**		**8**	0.014	0.001	**9**	0.009	0.001
		statistical	unpaired T-test, * P=0.0270					
	**22.6 kHz**		**8**	0.010	0.002	**9**	0.007	0.002
		statistical	unpaired T-test, ns P=0.2770					
	**32 kHz**		**8**	0.005	0.002	**9**	0.002	0.001
		statistical	unpaired T-test, ns P=0.1669					

Table S18

Figure S4A-L

			Slope wave I amplitude					
			Pex1 $^{fl/fl}$			*VGlut3 $^{cre/cre}$ Pex1 $^{fl/fl}$*		
			n	Mean	S.E.M.	n	Mean	S.E.M.
1 month	**5.6 kHz**		**16**	0.012	0.001	**12**	0.008	0.001
		statistical test	unpaired T-test, * P=0.0174					
	**8 kHz**		**16**	0.016	0.001	**12**	0.011	0.001
		statistical test	unpaired T-test, * P=0.0175					
	**11.3 kHz**		**16**	0.020	0.002	**12**	0.015	0.001
		statistical test	unpaired T-test, * P=0.0283					
	**16 kHz**		**16**	0.017	0.001	**12**	0.013	0.001
		statistical test	unpaired T-test, * P=0.00265					
	**22.6 kHz**		**16**	0.014	0.001	**12**	0.010	0.001
		statistical test	unpaired T-test, ns P=0.0668					
	**32 kHz**		**16**	0.010	0.001	**11**	0.007	0.000
		statistical test	unpaired T-test, ns P=0.0544					
4 months	**5.6 kHz**		**8**	0.008	0.001	**9**	0.005	0.000
		statistical test	unpaired T-test, *** P=0.0007					
	**8 kHz**		**8**	0.011	0.001	**9**	0.007	0.001
		statistical test	unpaired T-test, ** P=0.0014					
	**11.3 kHz**		**8**	0.016	0.002	**9**	0.008	0.000
		statistical test	unpaired T-test, ** P=0.0028					
	**16 kHz**		**8**	0.011	0.001	**9**	0.007	0.001
		statistical test	unpaired T-test, ** P=0.0040					
	**22.6 kHz**		**8**	0.010	0.001	**9**	0.006	0.000
		statistical test	unpaired T-test, * P=0.0101					
	**32 kHz**		**8**	0.006	0.001	**9**	0.005	0.000
		statistical test	unpaired T-test, ns P=0.0807					

Table S19

Figure S6A

		% of matched ribbon synapse/IHC							
		Pex1 fl/fl				*VGlut3 cre/cre Pex1 fl/fl*			
		n (mice)	n (IHC)	Mean	S.E.M.	n (mice)	n (IHC)	Mean	S.E.M.
8 kHz		**6**	**21**	100.90	1.39	**6**	**23**	101.80	1.40
	statistical test	2way ANOVA Bonferroni's multicomparison test, ns P>0.999							
22.6 kHz		**4**	**14**	100.10	2.04	**6**	**22**	101.90	1.41
	statistical test	2way ANOVA Bonferroni's multicomparison test, ns P>0.999							

Figure S6B

		% CtBP2 puncta _ 8 KHz							
		Modiolar				Pillar			
		n (mice)	n (IHC)	Mean	S.E.M.	n (mice)	n (IHC)	Mean	S.E.M.
Pex1 fl/fl		**5**	**20**	54.43	3.00	**5**	**20**	45.57	2.98
	statistical test	unpaired t-test, **<u>* P=0.0420</u>**							
VGlut3 cre/cre Pex1 fl/fl		**4**	**15**	61.05	5.33	**4**	**15**	38.96	5.33
	statistical test	unpaired t-test , **<u>** P=0.0066</u>**							

Figure S6C

		% CtBP2 puncta _ 22.6 KHz							
		Modiolar				Pillar			
		n (mice)	n (IHC)	Mean	S.E.M.	n (mice)	n (IHC)	Mean	S.E.M.
Pex1 fl/fl		**4**	**15**	53.37	3.03	**4**	**15**	46.63	3.65
	statistical test	unpaired t-test , ns P=0.1272							
VGlut3 cre/cre Pex1 fl/fl		**4**	**12**	55.91	3.65	**4**	**12**	44.10	3.65
	statistical test	unpaired t-test , **<u>* P=0.0320</u>**							
